# Urinary Metabolomic Analysis of Prostate Cancer by UPLC-FTMS and UPLC-Ion Trap MS^n^

**DOI:** 10.3390/diagnostics13132270

**Published:** 2023-07-04

**Authors:** Chien-Lun Chen, Yi-Ting Chen, Wen-Yu Liao, Yu-Sun Chang, Jau-Song Yu, Bao-Rong Juo

**Affiliations:** 1 Molecular Medicine Research Center, Chang Gung University, Kwei-San, Taoyuan 33302, Taiwan; 2 Department of Urology, Chang Gung Memorial Hospital, College of Medicine, Chang Gung University, Kwei-San, Taoyuan 33305, Taiwan; 3 Department of Biomedical Sciences, College of Medicine, Chang Gung University, Taoyuan 33302, Taiwan; 4 Department of Cell and Molecular Biology, College of Medicine, Chang Gung University, Kwei-San, Taoyuan 33302, Taiwan

**Keywords:** urinary metabolomics, prostate cancer, biomarker

## Abstract

Accumulative evidence suggests metabolic disorders correlate with prostate cancer. Metabolic profiling of urine allows the measurement of numerous metabolites simultaneously. This study set up a metabolomic platform consisting of UPLC-FTMS and UPLC-ion trap MS^n^ for urine metabolome analysis. The platform improved retention time, mass accuracy, and signal stability. Additionally, the product ion spectrum obtained from ion trap MS^n^ facilitated structure elucidation of candidate metabolites, especially when authentic standards were not available. Urine samples from six hernia patients and six BPH patients were used for the initial establishment of the analytic platform. This platform was further employed to analyze the urine samples of 27 PCa and 49 BPH patients. Choosing the upper and lower 16% of metabolites, 258 metabolite candidates were selected. Twenty-four of them with AUC values larger than 0.65 were further selected. Eighteen of the twenty-four features can be matched in METLIN and HMDB. Eleven of the eighteen features can be interpreted by MS^n^ experiments. They were used for the combination achieving the best differential power. Finally, four metabolites were combined to reach the AUC value of 0.842 (CI 95, 0.7559 to 0.9279). This study demonstrates the urinary metabolomic analysis of prostate cancer and sheds light on future research.

## 1. Introduction

According to Globocan 2020, prostate cancer is the most frequently diagnosed cancer and the second leading cause of cancer death in men. There were an estimated 1.4 million new cases of prostate cancer worldwide in 2020 [1]. Prostate cancer tends to develop in men aged 65 and over [2]. It has been shown that metabolic syndrome and obesity correlate with benign prostatic hyperplasia (BPH) and prostate cancer (PCa) [3]. Metabolic analysis of prostate disease is urgently needed.

Prostate cells possess a unique metabolism specialized for producing, storing, and secreting PSA, polyamines, and citrate [4,5,6]. PCa is distinguishable from BPH and normal prostate by its high lactate and low citrate levels; hence, metabolomic analysis is ideal for studying the disease [7,8,9,10,11,12,13,14].

Various analytic platforms have been used for metabolomic analyses [9,10,15,16,17,18,19]. However, nuclear magnetic resonance (NMR) spectroscopy and mass spectrometry (MS) are the most common tools. The advantage of MS methods over NMR is much higher sensitivity and detection of metabolites at much lower concentrations. Moreover, the acquired sample information provided by MS is specific to each metabolite, while NMR provides information about the chemical structure and quantitative information about metabolite concentrations.

MS is generally hyphenated with gas or liquid chromatography (GC, LC). For GC-MS analysis, derivatization is important to improve the volatility of metabolites. Sometimes, derivatization can also result in metabolite degradation [19]. LC-MS does not need derivatization and is widely applied in metabolomic analysis. Due to the sample complexities of metabolomic analysis, analysis by conventional LC was swiftly superseded by ultra-performance liquid chromatography (UPLC). UPLC equipped with a sub-2-μm column is acclaimed for its great resolution, high sensitivity, and rapid analytical speed [20,21]. For metabolomic analysis, Q-Tof or Tof analyzers are commonly utilized to match the fast speed of UPLC [22,23]. Though the accuracy of Q-Tof and Tof MS are listed in the range of 1~5 ppm [24], it was found that errors could be enlarged. Sometimes, the error was as significant as 20 ppm in the metabolomic analysis [25]. However, the first step for chemical structural identification of metabolites using database searching needs accurate results to remove false positive candidates. Fourier transform mass spectrometry (FTMS) is an accurate and stable analyzer. The analyzer is shielded by an isothermal shell and provides high resolution and highly sensitive detection. The accuracy is generally in the range of 1.5 to sub-ppm. These characteristics facilitate the first step of metabolite identification in the metabolomic analysis.

Generally, two independent and orthogonal data related to an authentic compound analyzed under identical experimental conditions are proposed as a necessity to validate non-novel metabolite identifications [26]. In addition to accurate mass, a double check is essential when identifying metabolites. For some commercially available metabolites, identifications rely on matching unknown compounds with authentic standards on the retention and the accuracy mass. However, most of the time, many metabolites on the search list are unavailable. Therefore, another possibility for metabolite identification is performing a product ion experiment to identify the structure information if the authentic compound is unavailable.

If a metabolite has been identified only through a parent ion mass match to a database, its identity should only be considered “very tentative”. To confirm metabolite candidates, researchers are required to purchase a lot of standard compounds. On the other hand, if a compound has been identified by matching the retention time, parent ion mass, and mass fragment patterns of a known standard, the identification of this compound is confident. To avoid the need to purchase many standards, we employed MS^n^ to facilitate our protocol in two aspects. The first is to narrow down the candidate list before matching the endogenous metabolites using standard compounds, and the second is to provide more confidence for the metabolites if the authentic compounds are unavailable [27].

FTMS is not the best for MS^n^ experiments to analyze complex samples with a wide range of concentrations [28]. The fringe field of the high field magnet may reject large ions, and the high radio frequency of small product ions may result in poor trapping efficiency of small ions. These reasons make the sensitivity of the MS^n^ experiment of FTMS not satisfactory, especially for those low abundant and small or high molecular weight metabolites. To overcome these limitations, we employed an ion trap mass spectrometer as the analyzer for MS^n^ analysis. Ion trap MS has the ability of ion storage. Even though the ion is low abundant, it could be collected longer to accumulate enough ion signals [29]. The scanning speed of ion trap MS is faster than FTMS. Thus, the ion detection efficiency is better for ion trap MS in the low mass region. Additionally, the MS^n^ in ion trap MS could provide the structural information of metabolite candidates. Collectively, the sensitivity of the product ion spectrum from ion trap MS is better than those obtained from FTMS.

This study established the UPLC-FTMS and UPLC-ion trap MS^n^ that might better resolve and characterize metabolically related differences between PCa and BPH.

## 2. Materials and Methods

### 2.1. Sample Collection and Preparation

All urine samples were collected at Chang Gung Memorial Hospital, Taoyuan, Taiwan. The study protocol was approved by the Medical Ethics and Human Clinical Trial Committee at Chang Gung Memorial Hospital. All urine samples were collected on the first morning after admission and before any operation. As the normal range of urine specific gravity is 1.003~1.030, urine samples were discarded if out of this range. All prostate cancer patients were diagnosed before the commencement of androgen deprivation or any focal therapy such as radiation. For the concern of metabolic interference, any BPH patient exposed to 5-α-reductase inhibitor was also excluded from this study. The collection, processing, and storage of urine samples were described in our previous publication [30]. Briefly, the urine was collected in the presence of a protease inhibitor cocktail tablet (one tablet per 50 mL urine) and sodium azide (1 mM). Removal of cells and debris by centrifugation (5000× *g* for 30 min at 4 °C) was carried out within one hour after sample collection. The sample was then kept at −80 °C for long-term storage. Before MS analysis, the sample was thawed at 4 °C. Freezing point depression was measured to determine the osmolarities of samples using a model 3320 osmometer (Advanced Instruments, Norwood, MA, USA). In total, 50 μL of urine were diluted four times with methanol and centrifuged at 13,200× *g* for 15 min at 4 °C. The supernatant was dried under N_2_. The sample was re-dissolved with 50 μL solvent consisting of MeOH: H_2_O = 2:1. The sample was centrifuged at 13,200× *g* for 15 min at 4 °C again; the supernatant was collected and used for LC-MS analysis.

In the initial establishment of the metabolomic analysis, the urine samples from 6 hernia patients (age 50~68, mean 62.7) and 6 BPH patients (age 64~77, mean 68.3) were used. They were all male with a comparable age range. This platform was further employed to differentiate the metabolomic profile of the prostatic disease between BPH and prostate cancer.

### 2.2. Chemicals

Protease inhibitor cocktail tablets were purchased from Roche (Mannheim, Germany). Solvents for the sample preparation and LC-MS analysis, such as methanol, water, acetonitrile with 0.1% formic acid, and water with 0.1% formic acid, were purchased from Sigma–Aldrich (St. Louis, MO, USA). These solvents were of CHROMASOLV grade. N_2_, N_2_-Dimethylguanosine, 7-Ketodeoxycholic acid, and Phenylacetyl L-glutamine were purchased from Toronto Research Chemicals Inc. (North York, ON, Canada). The other chemicals were all purchased from Sigma–Aldrich (St. Louis, MO, USA). The peptide was synthesized by Kelowna International Scientific Inc. (Kaohsiung, Taiwan).

### 2.3. Instrumentation

A UPLC system (Waters, Milford, MA, USA) equipped with a C18 reverse phase column (2.1 × 100 mm, 1.8 μm, HSS-T3, Waters, Milford, MA, USA) was coupled with either an FT-ICR MS (Apex-Q 94, Bruker Daltonics, Billerica, MA, USA) or an ion trap MS (HCT, Bruker Daltonics, Billerica, MA, USA) in the positive ESI mode. The flow rate was 0.1 mL/min at the beginning (99% solvent A: 0.1% formic acid; 1% solvent B: acetonitrile with 0.1% formic acid). One μL sample was injected. After injection, 1% solvent B was held for 5 min and increased to 50% in 9 min, then to 90% in 6 min, and 99% in 12 min held for 2 min, and the flow rate was increased to 0.5 mL/min, 5 min later. The flow rate was then reduced to 0.1 mL/min and back to 1% in 0.1 min and held for 7 min.

The mass spectra were recorded using the full scan mode, covering a mass range of *m*/*z* 100–1000. The sample size was 256 k, the calibration standard was 10 mM sodium formate in isopropanol: H_2_O = 9:1. The resolution was 17,944 at *m*/*z* 430.9138 (the ion formula is Na(NaCOOH)_6_), the source accumulation time was 0.01 s, and the collision cell time was 0.08 s. The capillary voltage was set to 4200 V, and the spray shield voltage was set to 3500 V. The nebulizing gas and drying gas flow were set to 4.0 and 4.6 L/min, respectively. The drying temperature was 190 °C.

An equal amount of each sample in the analysis was mixed to be the QC sample. Before batch analysis, the QC sample was injected 10 times for the condition of the UPLC column, and the sample injection sequence was randomized to reduce the effect of contamination from the previous injections. The sample injection sequence was randomized according to the suggestions of Wang EJ et al. [31]. In the injection sequence, every 10 analyses, the QC sample was injected to check the system stability throughout the whole analysis.

### 2.4. Data Analysis

Principal component analysis (PCA) and *t*-test analyses were performed by the software of ProfileAnalysis 2.0 (Bruker Daltonics, Billerica, MA, USA). The MS data from 0.5 to 17 min were used for analysis. The advanced bucket for time and mass was set to 0.3 s and 5 mDa, respectively. The S/N threshold was set to 5 under the “calculate compound” mode. The raw data of LC-MS was imported into the ProfileAnalysis, and PCA analysis was performed first. Additionally, the “count number” filter was applied. The “count number” means the encounters of a feature detected in the sample batch. It is used for reducing the low confident features. In this study, about half number of one group sample was used. If the sample numbers were different for different sample groups, the smallest sample number was selected to set the criteria. Then, the features were filtered by the “count number”, and the *t*-test calculation was performed. All tests were two-sided, and a *p*-value less than 0.05 was considered statistically significant.

### 2.5. Normalization and Significance Criteria

The numbers of detected features vs. their log (ratio) plot were drawn for the evaluation of the need for normalization. By fitting the curve with Gaussian distribution, the SD and shift (x_0_) were determined. If the x_0_ to 0 was less than |±0.04|it meant that the error of ratio was smaller than 10%, and the normalization was not processed. Using 0 ± 1 SD as the criteria, the top and bottom 16% features were selected for further analysis.

### 2.6. Web Searching

The mass list was searched against two web databases, the METLIN and the HMDB databases [32,33,34]. The mass tolerance is 5 ppm for METLIN and 0.01 Dalton for HMDB. The formulas of candidates obtained from the search were compared with the calculated isotope pattern of the corresponding feature. The matched candidates were further investigated by MS^2^ and/or MS^3^ experiments.

### 2.7. Statistical Analysis

Differences in concentration levels of urinary metabolites between different groups were analyzed using the nonparametric Mann–Whitney *U*-test. In addition, SPSS software was also used for statistical analyses of receiver operator characteristic (ROC) curves and areas under the curve (AUC) to check the abilities of metabolites to discriminate different groups.

## 3. Results

### 3.1. Workflow for Urinary Metabolomic Analysis by Integration of UPLC-FTMS and UPLC-Ion Trap

**Figure 1:**
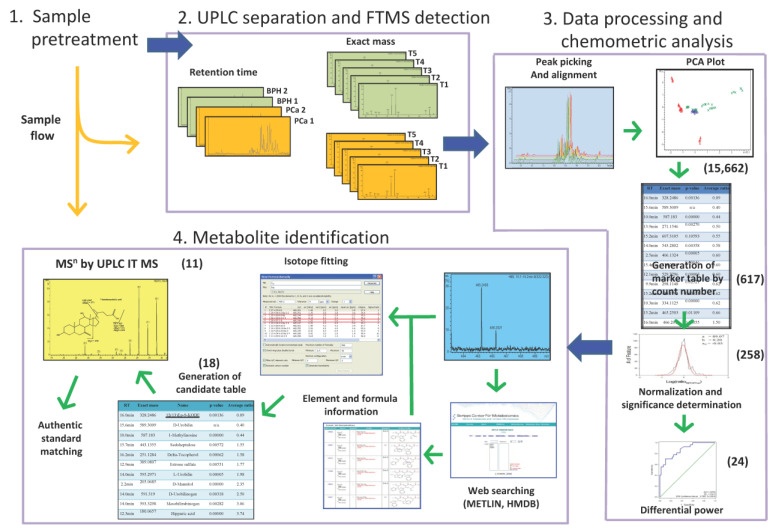
Workflow for urinary metabolomic analysis by integration of UPLC-FTMS and UPLC-ion trap. The numbers in the parenthesis indicated the feature numbers after the filtration by different criteria. Colors in the embedded image 2 indicate grouping during analysis.

Figure 1 demonstrates the MS-based metabolic analysis workflow for urine in this study. A UPLC system was employed for the separation of metabolites. The FTMS and ion trap MS^n^ (*n* = 2~3) were for the exact mass detection and structure determination, respectively.

### 3.2. Optimization of UPLC-FTMS System

FTMS is chiefly characterized by its high mass accuracy, high resolution, and high sensitivity, and these characteristics make it an excellent tool for small molecule detection. However, FTMS needs a longer time to collect stable signals (cycle time > 5 s) than other types of mass spectrometers; for example, the Q-Tof mass spectrometer can be operated at a speed of 0.1 s/scan [35]. If the signal collection time of FTMS is not long enough, the signal will be poor or fluctuate. When MS was hyphenated with chromatography, the first criterion for detecting a good peak shape is that the eluted peak width should be wide enough, and the MS can detect enough data points to describe the original peak shape. Generally, 10~20 data points for a peak are preferred.

The advantages of UPLC were recognized for its high speed and high resolution; moreover, we found that it also provided high accuracy in the retention time of peaks. Untargeted metabolomic analysis has the advantage of discovering novel metabolites. There is no internal standard added to the samples, and the analyzed results are directly compared to determine the difference. The analytical instruments must be consistent and accurate throughout the duration of the analysis in order to produce results that are comparable between sessions. In this circumstance, the stability of UPLC can help in the untargeted metabolomic analysis.

**Figure 2:**
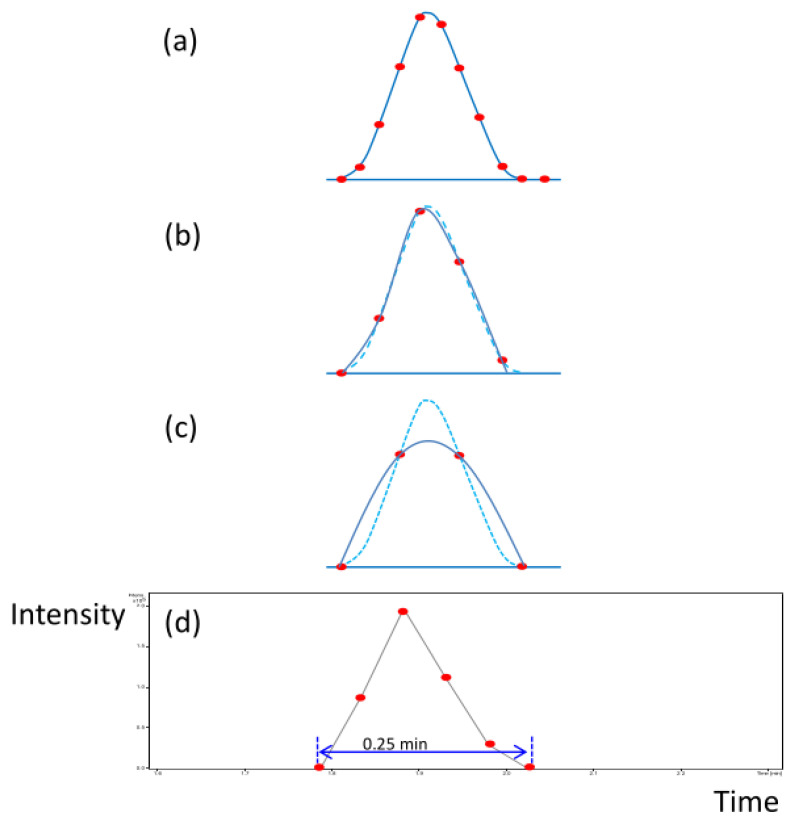
(**a**) A diagram of 11 data points describing a peak. According to the Savitzky–Golay smoothing filtering method, an LC peak needs at least 11 data points to describe its sharpness for quantitation. Ten data points are used for peak description and one point for the baseline determination. (**b**) When the data points are only five points for an LC peak, the data points can still keep the peak sharp, but the ability to find the shoulder peak will be lost. (**c**) If the data points are less than 5 points, for example, 4 data points, the peak will broaden, and the peak sensitivity will decrease after smoothing. The light blue dot line delineates the original sharp peak, and the dark blue solid line denotes the simulated peak after the data points are reduced. (**d**) An actual peak detected in our UPLC-FT MS system. Six data points were recorded for the LC peak.

In connecting UPLC to FTMS, the drawback was the incompatibility of analysis speed in common analytical conditions. For example, the peak width of UPLC is generally about 1.5~2.5 s (FWHM) in a flow rate of 0.6 mL/min on a 2.1 × 100 mm column, and the cycle time of FTMS is about 4.5 s at the resolution of 32,000 (512 k, single spectrum). According to the Savitzky–Golay smoothing filtering method, an LC peak needs at least 11 data points to describe its sharpness for quantitation [36]. Ten of the eleven data points are used for peak description, and one more for baseline determination (Figure 2a).

In the time frame of UPLC peaks, the FTMS should have a cycle time of less than 0.3~0.5 s. Nevertheless, in the time frame of FTMS, the separation speed of UPLC should slow down to make the LC peak wider than 45 s. ESI is known to be a concentration-dependent detector. When we increase the width of an LC peak by reducing the flow rate of LC, the peak height is inversely proportional to the peak width, and the signal of the mass spectrometer decreases with the peak height decreasing.

To resolve this challenge, we reduced the flow rate of UPLC to provide a longer time for FTMS scanning, speed up the scanning of FTMS to obtain more data points, and restrain the peak width from maintaining the sensitivity of ESI detection. The cycle time was reduced from 4.5 to 3 s/spectrum to speed up the scanning speed of FTMS. Under such conditions, 10 data points would take about 30 s to obtain a stable signal in the FTMS system; it took too long for the UPLC separation, and the mass sensitivity dropped significantly. Therefore, the data points were reduced to five to save the separation time. As illustrated in Figure 2b, when the data points of a peak were reduced to five points, the peak shape could be almost maintained, and the sensitivity would not be significantly reduced. If the data points were further reduced to four points, the peak shape would be distorted, and the sensitivity and resolution would be decreased (Figure 2c). The flow rate used for the 2.1 mm × 100 mm UPLC column was generally in the range of 0.4~0.6 mL/min, and the peak width was about 1.5~2.5 s (FWHM). After the flow rate adjustment, the flow rate was reduced to 0.1 mL/min to widen the peak width to 7.5 s (FWHM), and six data points could be detected for an ion with an intensity of 2 × 105 counts in our system (Figure 2d), which could provide a minimum data point for describing the sharpness of LC peak.

As suggested by the Savitzky–Golay smoothing filtering method, when the data point was less than 10, the shoulder peak could not be drawn perfectly, and the quantitation might have problems. However, because of the high resolving power of FTMS, the high mass resolution could help in separating two overlapping peaks in LC (in the time domain) into two distinct peaks in MS (in the mass domain). Only when the co-eluted metabolites are structural isomers, the quantitation will fail. Additionally, UPLC is also a high-resolution instrument. Poor separation for a UPLC is minor compared to that of a conventional LC. All the features detected in the metabolomic analysis are currently checked manually for overlaps.

### 3.3. Analysis of Urine Samples from BPH and Hernia Patients

Urine samples from six BPH and six hernia patients were used to test the platform. In this analysis batch, 79 analyses (6 BPH samples × 6 injections, 6 hernia samples × 6 injections, and 7 QC runs) were run after the system was conditioned with 10 QC samples.

**Figure 3:**
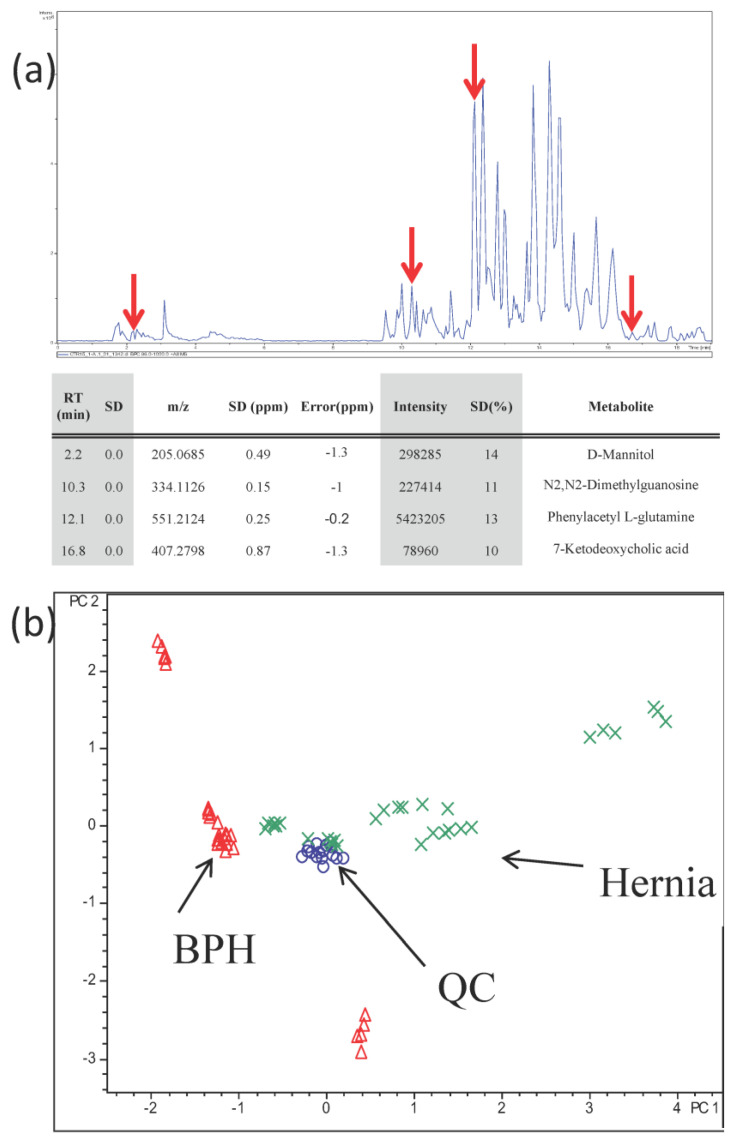
(**a**) A typical base peak chromatogram of BPH urine. The four arrows indicate the retention times of four selected features. They were *m*/*z* 205.0685, 334.1126, 551.2124, and 407.2798 at retention times of 2.2, 10.3, 12.1, and 16.8 min, respectively. These four features are different in retention times, molecular weights, and ion intensities. They were used to evaluate the stability of the UPLC-FTMS. They were finally identified as D-mannitol, N_2_, N_2_-dimethylguanosine, phenylacetyl L-glutamine, and 7-ketodeoxycholic acid, respectively. The detected parameters are listed. (**b**) The PCA plot of BPH and Her urines. The symbol (Δ) denotes BPH, (x) Her, and (O) QC. The QC was made by mixing an equal amount of each urine sample.

A typical base peak chromatogram of BPH urine is shown in Figure 3a. Generally, the urine metabolites will be separated into two groups in the LC analysis. One group was eluted at about 1.8~6.0 min; the other group was eluted at 9.5~19.0 min. To evaluate the performance of the UPLC-FTMS platform, seven QC runs in the analysis sequence were used to check the data qualities. In the QC samples, we selected four features at different retention times with different mass values and intensities. They were *m*/*z* 205.0685, 334.1126, 551.2124, and 407.2798 ions at retention times of 2.2, 10.3, 12.1, and 16.8 min, respectively (Figure 3a). Their retention times were very accurate, their standard deviations were all 0.0 min, and the standard deviations of detected mass values were less than 0.9 ppm. Moreover, their standard deviations of intensities were all less than 14%. These four features were later identified as D-mannitol, N_2_, N_2_-dimethylguanosine, phenylacetyl L-glutamine, and 7-ketodeoxycholic acid (Supplementary Table S3), respectively. Their actual mass errors were all less than 1.3 ppm. The results indicate that our metabolomic analysis system was reliable and accurate within the analysis batch.

The principal component analysis (PCA) plot for the 79 analyses is shown in Figure 3b. Each point in the PCA plot represents one analysis of a sample. It appeared that all the QC samples were clustered together, demonstrating that the analysis conditions were consistent and reproducible throughout the whole analysis time. A clear separation of BPH and Her sample groups could be observed. Additionally, for each sample, although the sample injection sequence had been randomized, the six analytical replicates for each sample were still clustered closely in the plot. The results further demonstrated that our platform is stable and reliable for urinary metabolomic analysis.

After peak alignment, 17,708 features were detected in the PCA analysis. To remove the non-reproducible signals, we set the filter to half of the lower limit of the sample number for feature detection. In this analysis, the total analysis numbers of BPH and Her samples were all 36 (6 samples × 6 injections); therefore, the filter was set to 18. After filtration, 1010 features were detected.

First, the resolution of peak separation was manually checked to determine if the reduction of MS data points would affect the quality of LC-MS separation in the view of extracted ion chromatogram. Among the 1010 features, 56 features (5.5% of features) were found to be not baseline separated in their extracted ion chromatograms. The error rate for the relative quantitation experiment is about 5.5%. To avoid false positive results, we suggested that when potential markers were found, it was necessary to recheck the extracted ion chromatograms of the markers; then, the errors for quantitation could be largely reduced.

### 3.4. Analysis of Urine Samples from PCa and BPH Patients

**Figure 4:**
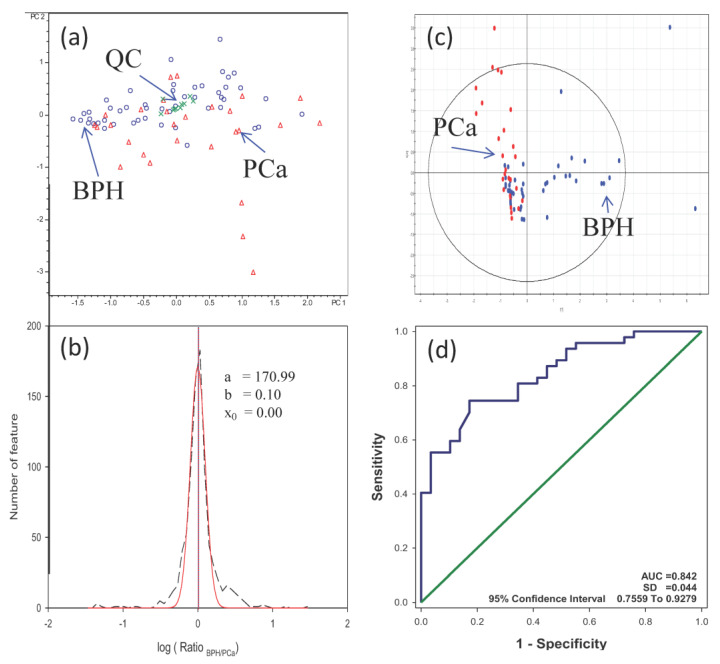
(**a**) The PCA plot of 27 PCa and 49 BPH urine samples. The symbol (Δ) denotes PCa, (O) BPH, and (Χ) QC. (**b**) The ratio distribution of 441 features. The distribution was fitted by the Gaussian distribution Curve; the three parameters were a: 170.99, b: 0.10, and x_0_: −0.0008. b was equal to one standard deviation of the curve, and x_0_ was the shift of the fitted curve to 0 (log1); (**c**) the reconstructed PCA plot made by four metabolites in PCa and BPH urines. The red spots denote PCa, and the blue spots denote BPH. (**d**) The ROC curve was calculated by the combination of four metabolites.

Using the platform established as described above, we further analyzed urine samples from 27 PCa and 49 BPH patients for the discovery of differential metabolites. The demographic data of the patients enrolled in this study are shown in Supplementary Table S1a,b. According to the suggestion of the Chemical Analysis Working Group, biological replicates are preferred over analytical replicates, as biological variance consistently exceeds analytical variance [26]. The sample number for each group in the study was more than 27 cases, and our metabolomic platform has been shown to be highly stable; thus, each sample was analyzed once. Figure 4a shows the PCA plot of these analyses. Again, all the QC samples were clustered together in this analysis which helped us to confirm the system stability. However, the separation of BPH and PCa samples in the PCA plot was not differentiated clearly.

A total of 15,662 features were detected in the PCA analysis. Again, the counted number of features was set to half of the PCa sample number. In this study, each sample was evaluated once. Hence, the counted number of features filter was set to 14. Following filtration, 617 features were identified, and a *t*-test was performed on these features.

Figure 4b is the plot of feature number vs. the log ratio_BPH/PCa_. The curve was fitted with Gaussian distribution. The x_0_ was −0.0008, which meant that the shift in ratio_BPH/PCa_ was less than 0.2%. According to the experiment results, data were not necessary to carry out the normalization. In the analysis, the b parameter was 0.10, equal to the standard deviation (SD). ±1 SD was utilized to identify candidates with more significant fold changes. It was equal to the ratios of 1.2 or 0.8. The features with ratio_BPH/PCa_ either larger than 1.20 or smaller than 0.80 were chosen. A total of 258 features were selected. We further applied the ROC calculation to these features. By filtering with the medium differential power AUC value, we found 24 features with an AUC value larger than 0.65.

Even though the analysis was performed at high mass accuracy (<1.3 ppm), many chemically possible formulas could be obtained, especially in higher mass regions. A simple way to identify the candidates of features is to combine high mass accuracy data with database searching in general mass spectral databanks or biochemical databases. We selected two human-related databases, METLIN and HMDB [32,33,34], for database search, producing a shortlist of potential candidates. The list can be shortened further by applying isotope ratios and performing MS^n^ experiments. As shown in Figure 1, the first step of metabolite identification was to check the feature’s exact mass and the isotope pattern. The exact mass can be utilized to search against the database, and the isotope pattern could be used to calculate the metabolite formula. However, in the LC-MS analysis, the metabolites are very likely to form clusters or metal adducts, and different element compositions will affect the calculation results of the isotope pattern. If the database is too complicated, the search results will also be complicated for the high possibility of false positive matching. By choosing a suitable database, candidates for metabolites were selected in advance based on the database’s properties. Thus, the possibility of false positive identification will be reduced. Furthermore, the resulting list of candidates will focus on those highly relevant metabolites and reduce the complexity of the candidate list. After the web search, 18 out of 24 features can be matched to compounds collected in METLIN and/or HMDB databases.

**Figure 5:**
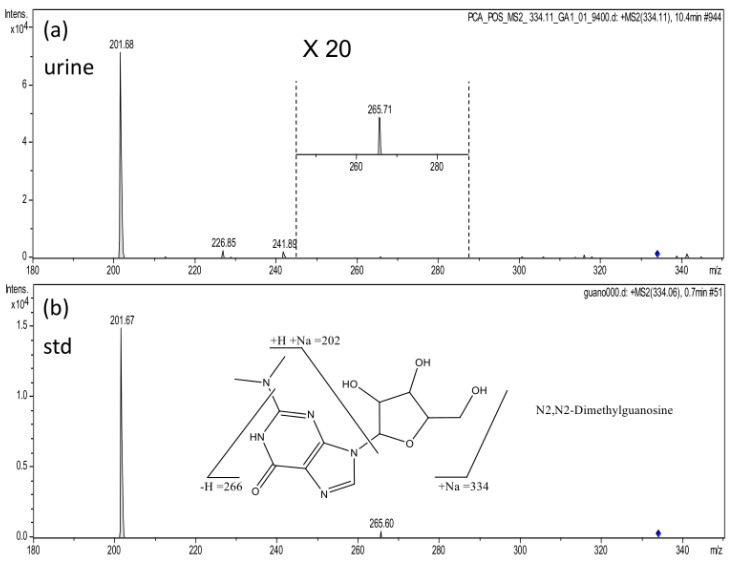
(**a**) The product ion spectrum of *m*/*z* 334.1 ion. (**b**) The product ion spectrum of N2, N2-dimethylganosine.

Among the 18 matched features, 11 can be interpreted by their product ion spectra (Supplementary Table S3). For example, a feature was detected at 10.4 min with *m*/*z* 334.1135. The mass value was used to search against the METLIN database. The search results showed that there were 10 possible candidates. By checking their formula, five categories of compounds and five element compositions were possible. They were imazethapyr (a pesticide) ([M + 2Na − H]^+^), 4-nitrophenol-alpha-D-galactopyranoside ([M + CH_3_OH + H]+), three sialic acid derivatives ([M + H-H_2_O]^+^), two dipeptides, and three nucleosides (one [M + ACN + Na]+ and two [M + Na]+). According to these formulas, the elements of C, H, O, N, and Na were used for calculating the possible isotope ratio of *m*/*z* 334.1135 ions. By matching the formulas obtained from the database search with the results from the isotope ratio calculation, we found that only three formulas (eight compounds) were possible. They were [M + − H_2_O]^+^, [M + ACN + Na]^+^, and [M + Na]^+^. We then performed an MS^2^ experiment to further explore the metabolite. As shown in Figure 5a, the fragment ions were very simple; only *m*/*z* 266, 202 ions were detected. Some clues can be found in the product ion spectrum: (1) no fragment ion was observed because of the loss of acetonitrile (Δm = 41); (2) the most abundant fragment ion resulted from the elimination of a fragment of mass 132. According to clue 1, the formula [M + ACN + Na]^+^, with solvent and sodium adducts, was not a possible formula. Two dipeptides and one nucleoside were excluded. In the product ion spectrum of *m*/*z* 344.11, no water loss was observed (Δm = 18). These sialic acid derivatives included five hydroxyl groups and lost water molecules readily during collision-induced dissociation (CID) processes. According to clue 2, we tentatively excluded the sialic acid derivatives.

Finally, two potential nucleosides remained, N_2_, N_2_-Dimethylguanosine and 1,7-Dimethylguanosine. Since they were all nucleosides, it made sense that these compounds may lose ribosyl moieties (Δm = 132) in their respective product ion spectra. By examining the fragment ions in the product ion spectrum of *m*/*z* 334.1134 in further detail, we identified an ion of mass 266. It was hypothesized that the ion would be generated by the breaking of the C–N bond on the side chain of N_2_-dimethylguanosine’s nucleobase. It appeared unlikely that the ion would result from the cleavage of 1, 7-dimethylguanosine. The identification of the metabolite as N_2_, N_2_-Dimethylguanosine was validated by comparison to its authentic standard (Figure 5b).

Table 1 lists the details of these 11 features (10 metabolites), and Table 2 lists the statistical results of the 10 metabolites. All 10 metabolites have medium differential power for differentiating the PCa and BPH patients. By further checking their *p*-values obtained from the *t*-test and fold change, there were seven metabolites with a *p*-value less than 0.05, and four of them were detected with a fold change of more than 2.0 or less than 0.52. All four metabolites have AUC values of more than 0.658. These four metabolites were used to reconstruct a new PCA plot. Figure 4c demonstrates that the BPH and PCa groups can be separated more clearly than their original PCA plot. The four metabolites were one dipeptide, a Vitamin D3 derivative, 7-Dehydropregnenolone, and L-Urobilinogen.

**Figure 6:**
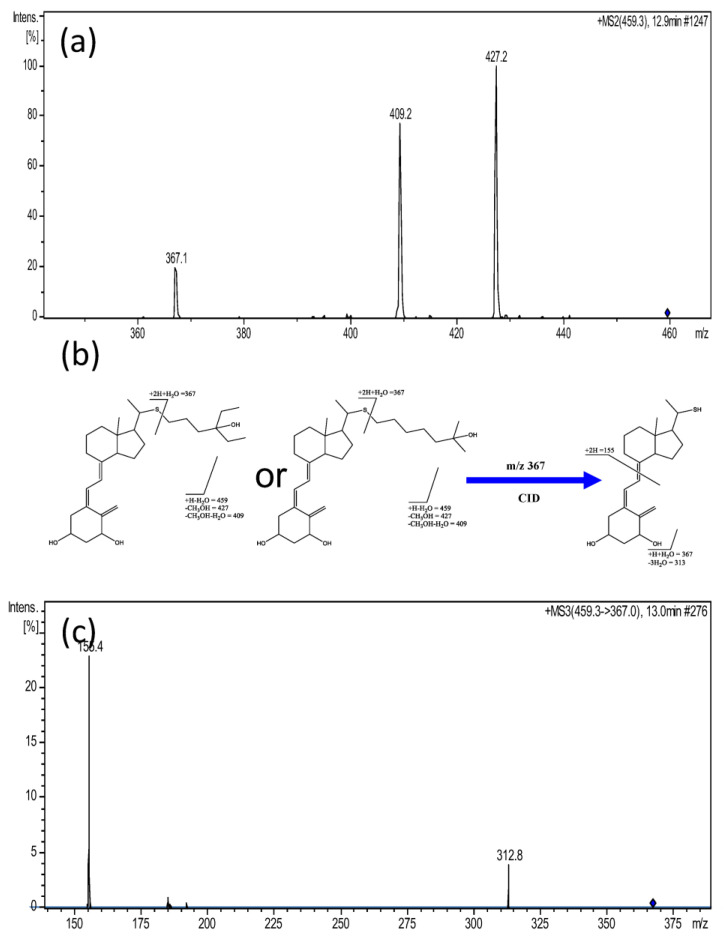
(**a**) The product ion spectrum of *m*/*z* 459.3 ion; (**b**) the interpretations of the fragmentation of *m*/*z* 459.3 ion and the fragmentation of the *m*/*z* 367.1 product ion. The chirality of molecular structure was not indicated. (**c**) The MS^3^ spectrum of *m*/*z* 459.3 to 367 ions. The *m*/*z* 459.3 ion was tentatively assigned as the Vitamin D3 derivative, but the side chain cannot be determined by MS^n^ experiments.

The dipeptide, Ala-Asp, was synthesized and confirmed by matching the retention time, accurate mass, and product ion spectrum (Supplementary Table S3). The standard compounds of the Vitamin D3 derivative, 7-Dehydropregnenolone and L-Urobilinogen, were unavailable. The Vitamin D3 derivative had four possible candidates. An MS^3^ experiment was then used to confirm the identity of the Vitamin D3 derivative. As shown in Figure 6a, the product ion spectrum of *m*/*z* 459 ions can be interpreted by the fragmentation of four possible Vitamin D3 derivatives (Figure 6b). They had different side chains and chiral centers. Unfortunately, there was no fragment ion with which the side chain structure could be confirmed. The *m*/*z* 367 ion was a hydronium adduct ion of the cleavage of Vitamin D3 derivative at the thioether bond. The *m*/*z* 367 ion in the product ion spectrum was selected for further fragmentation. The product ion spectrum of *m*/*z* 367 showed two fragment ions (Figure 6c); the *m*/*z* 313 ion resulted from losing three water molecules, and the *m*/*z* 155 ion from losing the fused ring. MS^2^ and MS^3^ experiments confirmed the skeleton of Vitamin D3, but the structure of its side chain and chirality could still not be determined by these MS^n^ experiments.

### 3.5. Clinical Narratives

Vitamin D (Vit D) deficiency has been linked to prostate cancer [37]. Sunlight exposure may boost the skin’s vitamin D production. Geographically, the incidence of prostate cancer is lowest in tropical zones, where exposure to sunshine is highest, compared to the subtropical and temperate zones [1]. Skin pigmentation influences cutaneous Vit D production. Black men in North America have a higher incidence of prostate cancer than white men.

Urobilinogen is a breakdown product of bilirubin, which is formed when red blood cells are broken down in the liver. Urobilinogen is then excreted in the urine and feces. The presence of urobilinogen in the urine can indicate liver or gallbladder problems, while the absence of urobilinogen in the urine can indicate a problem with red blood cell production or destruction. There is no direct relationship between urobilinogen and vitamin D. However, some liver disorders that affect the production of urobilinogen may also affect the absorption or metabolism of vitamin D. Therefore, it is important to monitor the levels of both urobilinogen and vitamin D in individuals with liver disorders. In the current study, it is intriguing to note that the urinary metabolites of vitamin D and urobilinogen are lower in PCa patients than in BPH patients (Table 2, BPH/PCa Ratio).

Cytochrome P450scc, in vitro or ex vivo in adrenal glands, cleaves the side chain of 7-dehydrocholesterol, producing 7-dehydropregnenolone. 7-dehydropregnenolone and its hydroxyderivatives can undergo UVB-induced transformation to corresponding pregnacalciferol and lumisterol-like compounds [38]. 7-dehydropregnenolone is a precursor to several hormones in the body. It can be converted into testosterone, which is important for male sexual development and function.

Combing the four metabolites, the AUC value reached 0.842 (CI 95, 0.7559 to 0.9279, Figure 4d). The value was in the middle–high range of the ROC analysis.

## 4. Conclusions

A platform comprised of UPLC-FTMS and UPLC-ion trap MS^n^ has been established. The platform is reliable and capable of conducting high-quality urinary metabolomic analysis. The identification of endogenous metabolites was more certain with MS^n^ prior to confirmation with authentic standards. The platform can also offer guidance for synthesizing the standards; not even the authentic standards were accessible. This study detected one peptide, one Vitamine D3 derivative, 7-dehydropregnenolone, and L-urobilinogen as potential biomarkers of prostate cancer. After combing the four metabolites, the AUC value reached 0.842 (CI 95, 0.7559 to 0.9279). The underlying metabolic pathway could elicit future investigation. Instead of a clinical approach, this study demonstrates urine metabolomic analysis of disease that could be an adjunct of disease diagnosis and shed light on future research.

## Figures and Tables

**Table 1:** The 10 metabolites identified in the PCa and BPH urines. The features were first selected with fold changes over 1.2 or less than 0.8, and the ratio criteria were determined by the ratio distribution plot. After applying the ROC calculation on the selected features, they were filtered with the medium differential power with an AUC value of 0.65, and then these features were used to search against the METLIN and HMDB databases. After the web searching, 18 out of 24 features can be matched to compounds collected in METLIN and/or HMDB databases. Among the 18 matched features, 11 features (10 metabolites) can be interpreted by their product ion spectra. #: metabolites; ^a^: the number of the product ion spectrum of the metabolite in Supplementary Table S3.

#	RT (min)	Exact Mass	Adduct Ion	Error (ppm)	Metlin ID	HMDB ID	Name	^a^ Spectrum
1	15.4	315.2365	[M + H ]^+^	14.9	x	HMDB13121	7-Dehydropregnenolone	1
2	11.7	318.1915	[M + CAN + H]^+^	3.1	66,195	x	p-Coumaroylagmatine	2
3	10.7	207.1107 185.1286	[M + CAN + Na] + [M + CAN + H]^+^	−1.2 0.9	6589	x	1-Aminocyclohexanecarboxylic acid	3 4
4	13.1	344.2436	[M + NH_4_]^+^	−1.6	36,202	x	2,3-dinor-11b-PGF2α	5
5	1.9	441.2987	[M + Na]^+^	2.8	41,996 41,998	x x	(24R)-1α,24-dihydroxy-22-oxacholecalciferol (24S)-1α,24-dihydroxy-22-oxacholecalciferol	6
6	10.4	334.1135	[M + Na]^+^	3.8	7086	HMDB04824	N2,N2-Dimethylguanosine	7
7	12.5	187.0225	[M + Na]^+^	0.7	44,770	x	Lumazine	8
8	14.9	679.4108	[M + 2ACN + H ]^+^	4.8	58,221	HMDB04157	L-Urobilinogen	9
9	12.7	459.3336	[M + H − H_2_O]^+^	−1.3	42,352 42,353 42,354 42,355	x x x x	1α,25-dihydroxy-24a-homo-26,27-dimethyl-22-thiacholecalciferol 1α,25-dihydroxy-26,27-dimethyl-24a-homo-22-thia-20-epicholecalciferol 1α,25-dihydroxy-24a,24b,24c-trihomo-22-thiacholecalciferol 1α,25-dihydroxy-24a,24b,24c-trihomo-22-thia-20-epicholecalciferol	10
10	12.5	227.0599	[M + Na]^+^	16.9	23,907	x	Ala Asp	11

**Table 2:** The statistics of the 10 metabolites in Table 1. The most abundant ion, the sodium adduct ion of 1-Aminocyclohexanecarboxylic acid, was used for the ratio calculation to achieve the best sensitivity.

#	Name	Ratio (BPH/PCa)	*p*	ROC Curve		
				AUC	SD	95% CI
1	7-Dehydropregnenolone	0.52	0.0207	0.685	0.067	0.5526 To 0.8171
2	p-Coumaroylagmatine	0.62	0.0662	0.677	0.065	0.5488 To 0.8042
3	1-Aminocyclohexanecarboxylic acid	0.66	0.00773	0.719	0.066	0.5902 To 0.8482
4	2,3-dinor-11b-PGF2α	0.74	0.12147	0.671	0.066	0.5413 To 0.8011
5	(24R)-1α,24-dihydroxy-22-oxacholecalciferol (24S)-1α,24-dihydroxy-22-oxacholecalciferol	0.76	0.00337	0.692	0.066	0.5636 To 0.8212
6	N2,N2-Dimethylguanosine	0.77	0.03143	0.697	0.067	0.5657 To 0.8281
7	Lumazine	1.54	0.05592	0.652	0.068	0.5186 To 0.7852
8	L-Urobilinogen	2.53	0.00006	0.716	0.057	0.6034 To 0.8282
9	1α,25-dihydroxy-24a-homo-26,27-dimethyl-22-thiacholecalciferol 1α,25-dihydroxy-26,27-dimethyl-24a-homo-22-thia-20-epicholecalciferol 1α,25-dihydroxy-24a,24b,24c-trihomo-22-thiacholecalciferol1α,25-dihydroxy-24a,24b,24c-trihomo-22-thia-20-epicholecalciferol	2.9	0.00983	0.658	0.061	0.5373 To 0.7779
10	Ala Asp	3.96	0.02371	0.697	0.059	0.5810 To 0.8121

## Data Availability

Not applicable.

## Supplementary Materials

The following supporting information can be downloaded at: https://www.mdpi.com/article/10.3390/diagnostics13132270/s1, Table S1a: Clinical data of 27 PCa patients and 49 BPH patients with analysis results of four metabolites; Table S1b: The demography of patients with benign prostatic hyperplasia (BPH) and prostate cancer (PCa); Table S2: The product ion spectra of metabolites in Table 1; Table S3: The product ion spectra of identified metabolites and their authentic standards.

## Abbreviations

AUC	Area Under Curve
BPH	Benign Prostatic Hyperplasia
CID	Collision-Induced Dissociation
ESI	Electrospray Ionization
FTMS	Fourier Transform Mass Spectrometry
FWHM	Full Width at Half Maximum
Her	Hernia
HMDB	The Human Metabolome Database
LC-MS	Liquid Chromatography-Mass Spectrometry
METLIN	The METLIN Metabolite and Chemical Entity Database (https://metlin.scripps.edu/landing_page.php?pgcontent=mainPage (accessed on 29 May 2023))
MS^n^	Mass Spectrometry in *n* (*n* = 2~3) rounds
NMR	Nuclear Magnetic Resonance
PCA	Principal Component Analysis
PCa	Prostate Cancer
QC	Quality Control
Q-Tof Quadruple Time-of-flight	
ROC curve	Receiver Operating Characteristic curve
UPLC	Ultra-Performance Liquid Chromatography
