# Supplementary material for: Urinary Metabolomic Analysis of Prostate Cancer by UPLC-FTMS and UPLC-Ion Trap MSn
Source: Diagnostics (Basel). 2023 Jul 4;13(13):2270. doi: 10.3390/diagnostics13132270 (PMC10340632; doi:10.3390/diagnostics13132270)
Supplement: Supplementary file 1 [file diagnostics-13-02270-s001.zip › diagnostics-2454829-Table S1.pdf]

Table S1a

Supplementary Table S1a Clinical data of 27 PCa patients and 49 BPH patients with analysis results of four metabolites

Patient	Age	Smoke	Hem	UTI	HTN	DM	Cr	Hb	Hepatitis	Dipeptide	Vit D	Urobilinogen	DHpregnenolone	iPSA	G	Stage
PCa01	59	0	0	0	1	0	0.88	13.7	1	0	0	0	141091	5.773	8	T3aN0M0

PCa02	68	1	0	0	1	0	0.99	12.1	0	0	0	0	47020	8.08	9	T3bN0Mx
PCa03	70	0	0	0	1	0	1.12	12	0	0	0	0	1100198	11.4	7	T3bN0M0
PCa04	67	0	0	0	1	0	0.86	12.2	1	0	46883	0	90653	17.13	9	T3bN0M0
PCa05	81	1	0	0	1	1	1.62	9	0	0	0	0	42607	12.738	7	T1cN0M0
PCa06	76	0	0	0	1	0	1.08	14.5	0	0	0	0	275199	10.86	8	T2cN0M0
PCa07	65	0	0	0	1	0	0.69	13.3	0	0	0	0	297848	2575	7	T4N1M1
PCa08	70	0	0	0	1	1	.0.86	9.5	1	0	0	0	166978	14.78	8	T2N0M0
PCa09	78	1	1	1	1	0	2.03	10.4	0	103483	0	0	78711	36.68	9	T3aN0M0
PCa10	68	0	0	0	1	0	1.14	14.3	0	0	0	0	622550	4.7	6	T2cN0M0
PCa11	63	0	0	0	0	0	0.99	13.6	0	29879	0	0	973039	6.51	6	T2cN0M0

PCa12	63	1	0	0	1	0	1.01	15	1	0	0	39785	380323	3.68	6	T2cN0M0
PCa13	66	0	1	0	1	1	-	13	1	0	0	0	403424	11.7	7	T2cN0M0
PCa14	76	0	0	0	1	1	1.5	8.2	0	0	0	0	58301	8433	8	TxNxM1
PCa15	84	0	0	0	0	0	0.88	11	0	0	0	0	77520	20.34	9	T2cN0M0
PCa16	58	0	0	0	1	-	0.85	14.2	1	0	0	0	234584	40.38	7	T3bN0M0
PCa17	76	0	0	0	0	0	0.83	13.6	-	0	0	0	301517	9	7	T2cN0M0
PCa18	56	0	0	0	0	0	0.73	14.5	1	0	0	0	134952	11.25	9	T3N0Mx
PCa19	73	1	0	0	0	0	0.89	13.9	0	0	0	0	0	11.58	7	T3bN1M1
PCa20	75	1	0	0	1	0	1.02	12.9	0	0	0	0	1330747	24.97	7	T4N1M1
PCa21	67	0	0	0	0	0	2.3	10	0	0	0	0	911468	175.53	9	T4N1M1

PCa22	65	0	0	0	0	0	0.88	11.9	0	0	45852	0	172578	8.3	7	T2N0M0
PCa23	67	1	-	-	0	0	0.62	9.6	1	0	61187	0	407458	197	9	TxNxM1
PCa24	70	1	0	0	0	0	0.77	14.8	0	0	0	39085	702634	17.32	6	T3aN0Mx
PCa25	64	0	0	0	0	0	1.22	11.9	0	0	0	0	294403	39.47	9	T3bN0M0
PCa26	80	1	1	1	0	0	1.31	13.7	0	0	0	0	346504	7	9	T3N0M0
PCa27	67	0	0	0	1	0	1	15.6	0	0	0	43566	120368	13.52	7	T3aN0M0
BPH01	64	0	0	0	0	0	1	13.3	0	128834	139006	115670	142431			
BPH02	65	1	0	0	1	1	1.2	18	0	343928	103151	193291	119447			
BPH03	65	0	0	0	1	0	1.01	13.7	0	373912	727377	136898	607633			

BPH04	70	0	0	1	1	0	4.55	12.5	0	141782<br>2	86371	254761	0
BPH05	77	0	0	0	0	0	0.87	14.9	0	211161	0	74858	0
BPH06	68	1	0	0	1	1	0.88	13.5	0	0	149650	101747	0
BPH07	66	-	0	0	0	0	1.78	-	0	99874	122342	102409	172022
BPH08	70	1	0	0	0	0	1.2	14	0	0	109583	96765	0
BPH09	73	-	0	0	1	0	10	15.5	0	169895	0	0	0
BPH10	74	-	0	0	0	0	-	-	1	274576	172688	204747	75826
BPH11	60	-	0	0	0	0	1	-	0	628921	144546	102714	112436
BPH12	55		0	0	0	0	-	-	0	0	198884	114044	655494

BPH13	69	1	0	0	0	0	0.83	12.4	0	455420	137815	88383	283130
BPH14	66	0	0	0	0	0	-	-	0	0	86646	86164	139071
BPH15	60	-	1	0	0	0	0.79	-	0	0	86263	89393	0
BPH16	64	-	0	0	0	0	-	-	0	393052	192179	202400	190525
BPH17	69	-	0	0	1	1	0.91	9.4	1	218141	130096	108352	294955
BPH18	70	-	0	0	1	0	1.52	13.5	0	0	0	22290	108977
BPH19	78	-	0	0	0	0	-	-	0	51541	0	0	213594
BPH20	58	0	0	0	1	1	-	14.2	0	36421	0	34352	209330
BPH21	72	-	0	0	0	0	1.26	-	0	0	0	0	0
BPH22	66	0	0	0	0	1	0.82	15.2	0	0	0	37592	75811

BPH23	69	0	0	0	0	0	0.98	13.9	0	0	0	0	119933
BPH24	63	1	0	0	1	0	1.23	14.5	0	0	0	0	247048
BPH25	60	0	0	0	1	0	0.96	15.1	0	0	0	0	207263
BPH26	79	-	0	1	0	0	0.99	-	0	40767	0	0	68688
BPH27	80	-	0	0	1	0	1.53	12.7	0	0	0	0	0
BPH28	76	1	0	0	0	1	0.76	10.3	0	0	0	0	79439
BPH29	61	-	0	0	1	1	0.77	16.8	0	0	0	0	153971
BPH30	53	-	0	0	0	0	2.71	13.4	0	0	0	0	217705
BPH31	52	0	0	0	1	0	0.95	15.6	0	0	0	0	314611
BPH32	49	-	0	1	0	0	1.1	-	0	0	0	0	0

BPH33	57	-	0	0	0	0	0.84	14.7	0	57700	0	40890	399714
BPH34	78	0	0	0	1	0	-	13.6	0	0	0	0	202547
BPH35	65	-	0	0	1	1	-	13.8	0	0	0	0	388267
BPH36	76	0	0	0	0	0	0.9	12.7	0	551293	66582	64191	224284
BPH37	57	0	0	0	0	0	0.9	14.3	0	28940	0	0	128074
BPH38	80	-	0	0	0	1	0.85	-	0	64742	0	201616	217989
BPH39	75	-	0	0	0	0	0.92	16.8	0	68484	60059	0	195436
BPH40	57	-	0	0	0	0	0.85	14.5	0	139129	0	0	0
BPH41	73	1	0	0	0	0	1.3	13.9	1	48552	59496	0	124555
BPH42	74	0	0	0	1	0	1.24	12.7	0	0	0	0	164663

BPH43	67	0	0	0	1	0	-	16.1	0	0	0	0	101485
BPH44	82	-	0	0	0	0	-	-	0	0	58572	0	147795
BPH45	58	0	0	0	1	0	0.97	15.5	1	0	0	0	136126
BPH46	68	0	0	0	0	0	-	13.5	0	0	0	23556	68927
BPH47	77	-	0	0	0	0	1.04	-	0	0	0	34829	58649
BPH48	56	0	0	0	1	0	0.9	17.2	0	0	0	0	124966
BPH49	83	1	1	1	1	1	0.89	12	0	0	0	48267	153731

PCa: Prostate cancer; BPH: Benign prostatic hyperpalsia; Hem: Hematuria; UTI: Urinary tract infection; HTN: Hypertension; DM: Diabetes Mellitus; Cr: Serum creatinine; Hb: Hemoglobin; iPSA: Initial PSA ; G: Gleason scoring; "0" : No; "1" : Yes; "-" : No Data.

Table S1b

Supplementary Table S1b The demography of patients with benign prostatic hyperplasia (BPH) and prostate cancer (PCa)

Characteristics	n (%)	BPH	PCa	*p*-valuess

Age at presentations				0.257
≧ 65 years	53(69.7)	32	21	
< 65 years	23 (30.3)	17	6	
Smoke				0.842
Yes	17(32.1)	8	9	
No	36(67.9)	18	18	
Hematuria				0.219
Yes	5(6.7)	2	3	
No	70(93.3)	47	23	
Urinary Tract Infection				0.943

Yes	6(8.0)	4	2	
No	69(92.0)	45	24	
Hypertension				0.123
Yes	36(47.4)	20	16	
No	40(52.6)	29	11	
Diabetes Mellitus				0.547
Yes	14(18.4)	10	4	
No	62(81.6)	39	23	
Renal insufficiency				1.000
Yes (Cr>= 1.5)	10(15.4)	6	4	

No	55(84.6)	33	22	
Anemia				0.087
Yes (Hb< 10)	5(8.1)	1	4	
No	57(91.9)	34	23	

Setting p<0.05 by Chi-square as significant difference statistically
