# Supplementary material for: Urinary Metabolomic Analysis of Prostate Cancer by UPLC-FTMS and UPLC-Ion Trap MSn
Source: Diagnostics (Basel). 2023 Jul 4;13(13):2270. doi: 10.3390/diagnostics13132270 (PMC10340632; doi:10.3390/diagnostics13132270)
Supplement: Supplementary file 1 [file diagnostics-13-02270-s001.zip › diagnostics-2454829-Table S2.pdf]

Supplementary Table S2. The product ion spectra of metabolites in the Table 1.

Spectrum Number	1	Detected Mass	315.2365
Adduct Formula	C 21 H 31 O 2		
Putative name	**NAME:** 7-Dehydropregnenolone		
Acc #	**Metlin:** x<br>**HMDB:**HMDB13121<br>**KEGG:**x		

Interpretation

7-Dehydropregnenolone

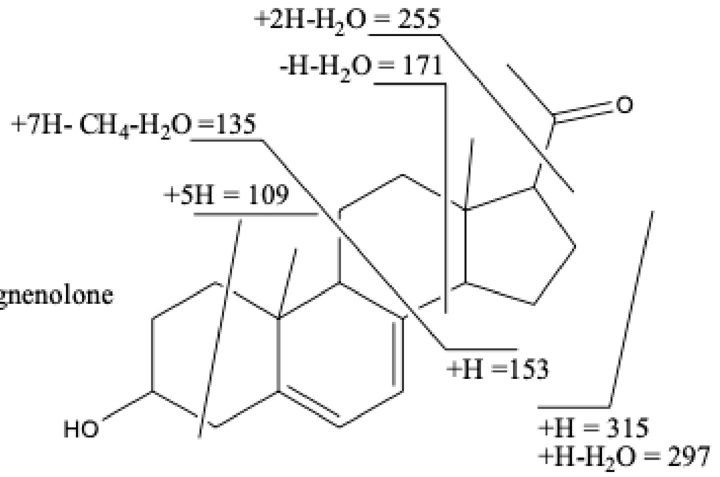

Product ion spectrum

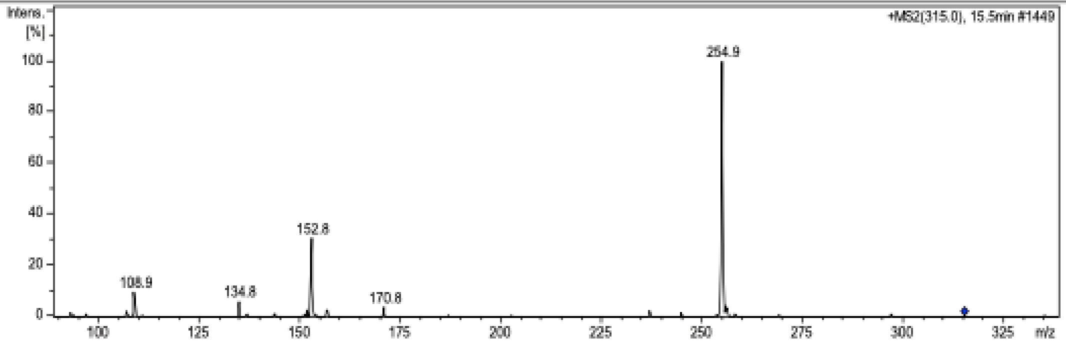

Spectrum Number	2	Detected Mass	318.1915
Adduct Formula	C 16 H 24 N 5 O 2		
Putative name	**NAME:** p-Coumaroylagmatine		
Acc #	**Metlin:** 66195<br>**HMDB:**x<br>**KEGG:**C04498		
Interpretation			

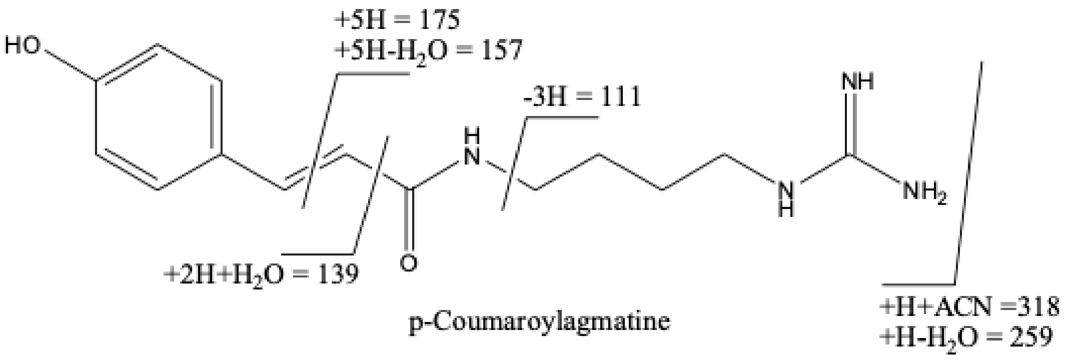

Product ion spectrum

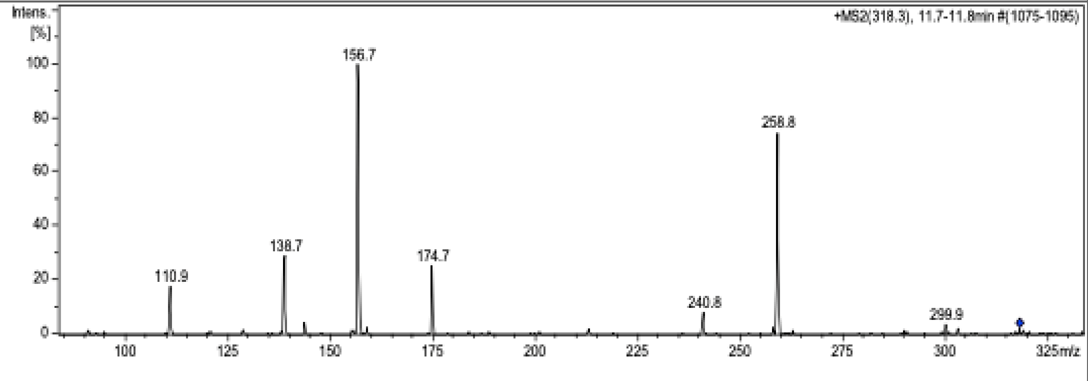

Spectrum Number	3	Detected Mass	185.1286
Adduct Formula	C9H17N2O2		
Putative name	**NAME:** 1-Aminocyclohexanecarboxylic acid		
Acc #	**Metlin:** 6589<br>**HMDB:**HMDB02279<br>**KEGG:**x		

Interpretation

1-Aminocyclohexanecarboxylic acid

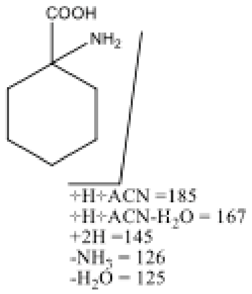

Product ion spectrum

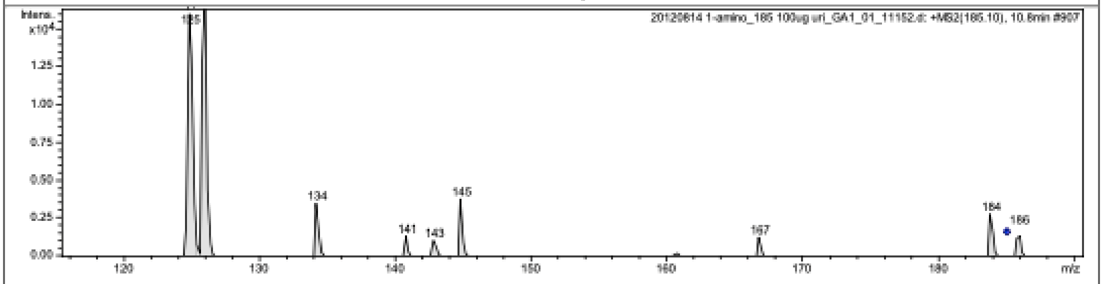

Spectrum Number	4	Detected Mass	207.1107
Adduct Formula	C 9 H 16 N 2 Na O 2		
Putative name	**NAME:** 1-Aminocyclohexanecarboxylic acid		
Acc #	**Metlin:** 6589<br>**HMDB:**HMDB02279<br>**KEGG:**x		

Interpretation

1-Aminocyclohexanecarboxylic acid

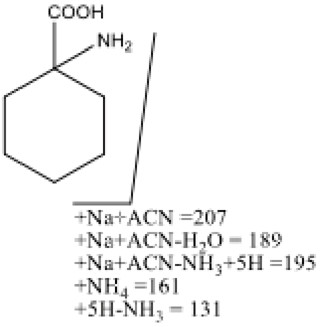

Product ion spectrum

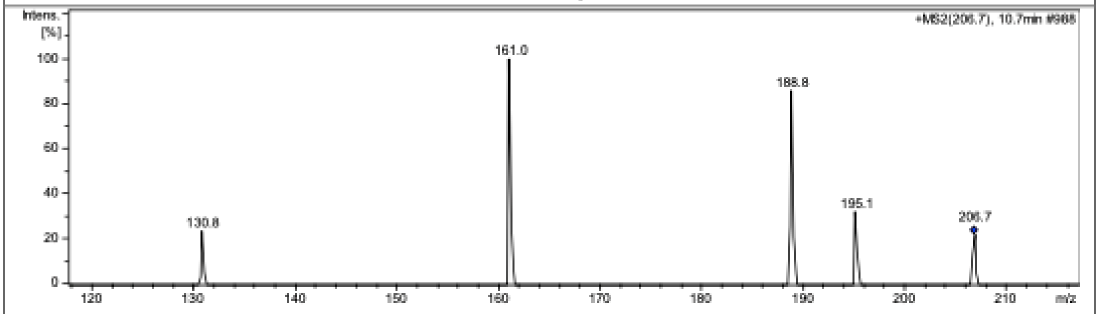

Spectrum Number	5	Detected Mass	344.2436
Adduct Formula	C 18 H 34 N O 5		
Putative name	**NAME:** 2,3-dinor-11b-PGF2α		
Acc #	**Metlin:** 36202<br>**HMDB:**x<br>**KEGG:**C14794		
Interpretation			

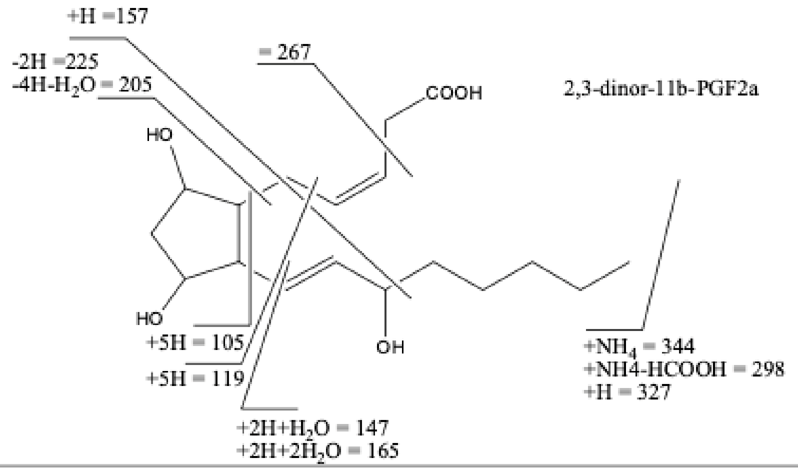

Product ion spectrum

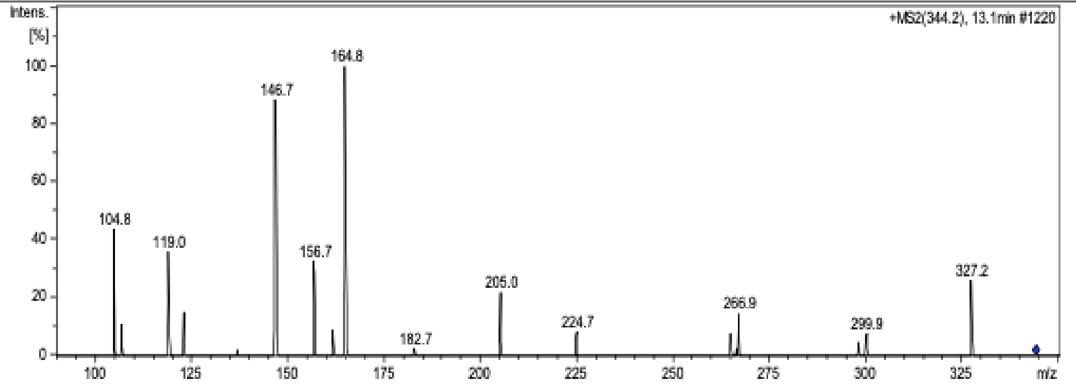

Spectrum Number	6	Detected Mass	441.2987
Adduct Formula	C 26 H 42 Na O 4		
Putative name	**NAME:** (24R)-1α,24-dihydroxy-22-oxacholecalciferol<br>(24S)-1α,24-dihydroxy-22-oxacholecalciferol		
Acc #	**Metlin:** 41996<br>41998<br>**HMDB:**x<br>**KEGG:**x		

Interpretation

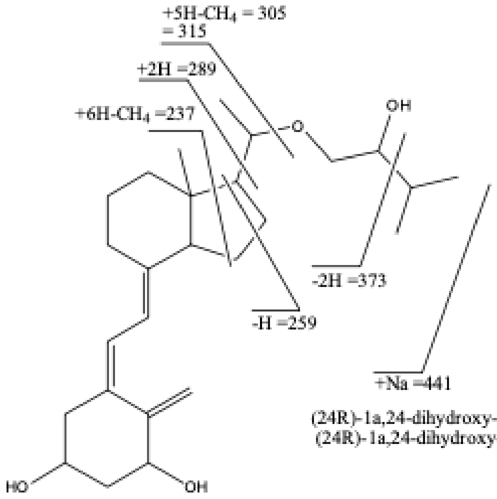

(24R)-1a,24-dihydroxy-22-oxavitamin D3 /
(24R)-1a,24-dihydroxy-22-oxacholecalciferol

Product ion spectrum

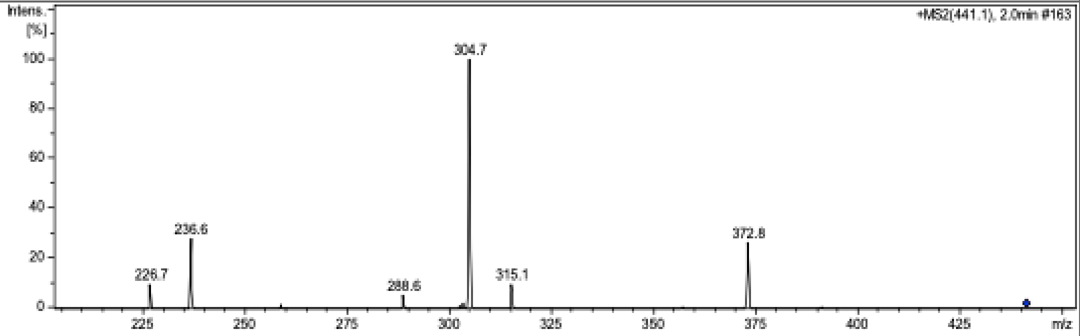

<table>
<tr><td>Spectrum Number</td><td>7</td><td>Detected Mass</td><td>334.1135</td></tr>
<tr><td>Adduct Formula</td><td colspan="3">C 12 H 17 N 5 Na O 5</td></tr>
<tr><td>Putative name</td><td colspan="3">NAME: N2,N2-Dimethylguanosine</td></tr>
<tr><td>Acc #</td><td colspan="3">Metlin: 7086
HMDB:HMDB04824
KEGG:x</td></tr>
<tr><td colspan="4">Interpretation</td></tr>
<tr><td colspan="4">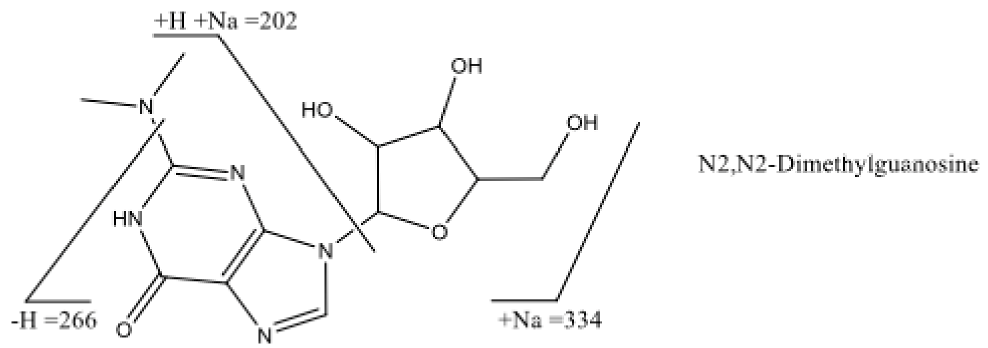
</td></tr>
<tr><td colspan="4">Product ion spectrum</td></tr>
<tr><td colspan="4">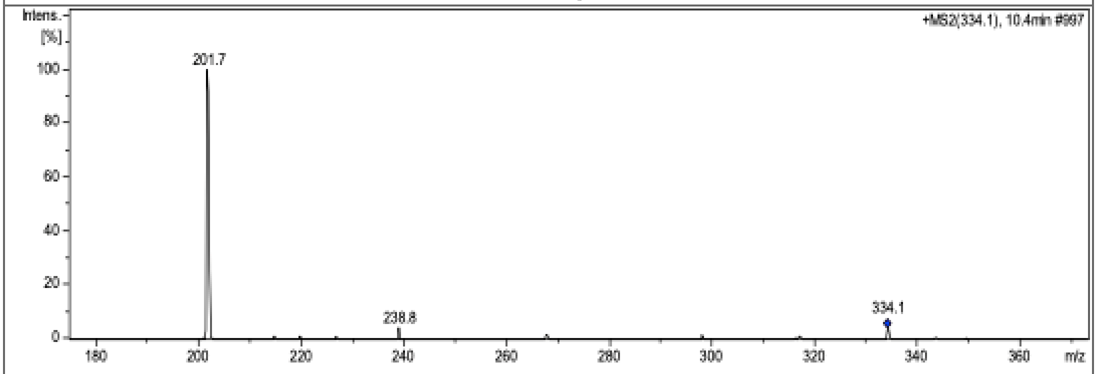
</td></tr>
</table>

Spectrum Number	8	Detected Mass	187.0225
Adduct Formula	C 6 H 4 N 4 Na O 2		
Putative name	**NAME:** Lumazine		
Acc #	**Metlin:** 44770<br>**HMDB:**x<br>**KEGG:**x		

Interpretation

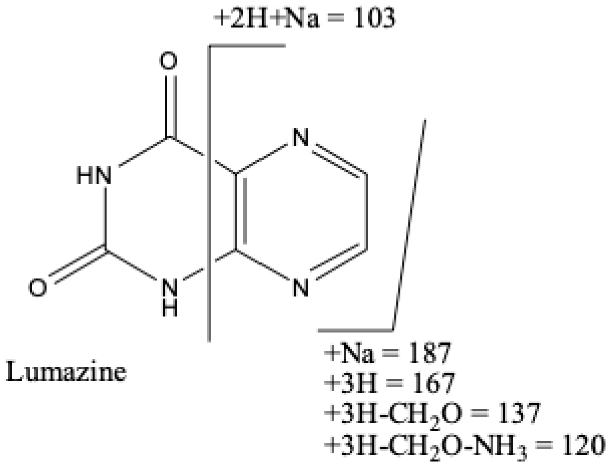

Product ion spectrum

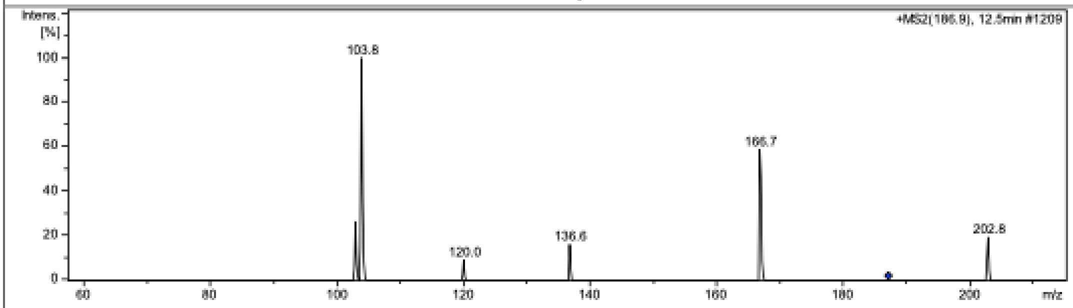

Spectrum Number	9	Detected Mass	679.4108
Adduct Formula	C 37 H 55 N 6 O 6		
Putative name	**NAME:** L-Urobilinogen		
Acc #	**Metlin:** x<br>**HMDB:**HMDB04157<br>**KEGG:**x		

Interpretation

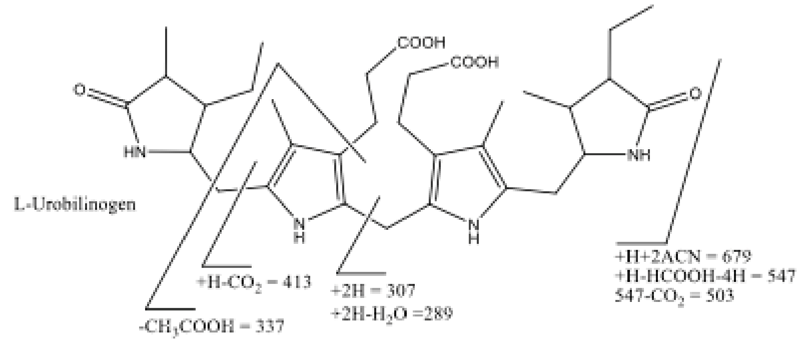

Product ion spectrum

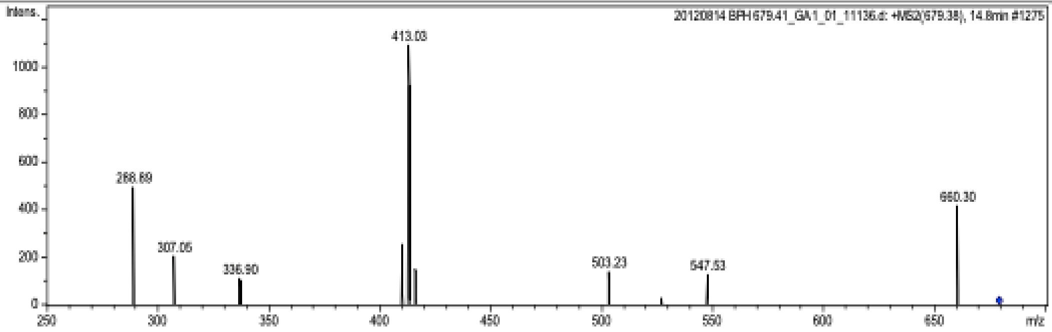

Spectrum Number	10	Detected Mass	459.3336
Adduct Formula	C 29 H 47 O 2 S		
Putative name	**NAME:** 1α,25-dihydroxy-24a-homo-26,27-dimethyl-22-thiavitamin D3<br>1α,25-dihydroxy-24a-homo-26,27-dimethyl-22-thiacholecalciferol<br>1α,25-dihydroxy-26,27-dimethyl-24a-homo-22-thia-20-epivitamin D3<br>1α,25-dihydroxy-26,27-dimethyl-24a-homo-22-thia-20-epicholecalcif		
Acc #	**Metlin:** 42352<br>42353<br>42354<br>42355<br>**HMDB:x**<br>**KEGG:x**		

Interpretation

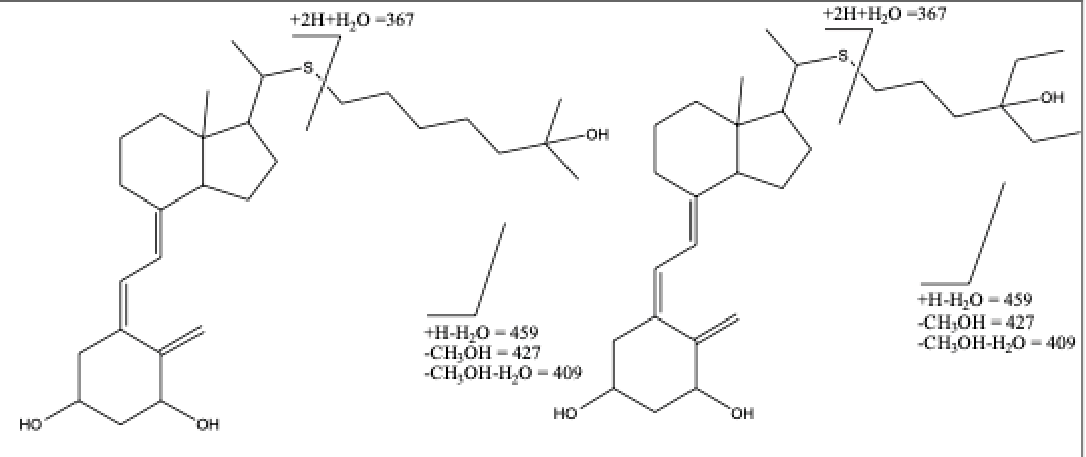

(5Z,7E)-(1S,3R)-26,27-dimethyl-24a-homo-9,10-seco-22-thia-5,7,10(19)-cholestatriene-1,3,25-triol
(5Z,7E)-(1S,3R)-24a,24b,24c-trihomo-9,10-seco-22-thia-5,7,10(19)-cholestatriene-1,3,25-triol

Product ion spectrum

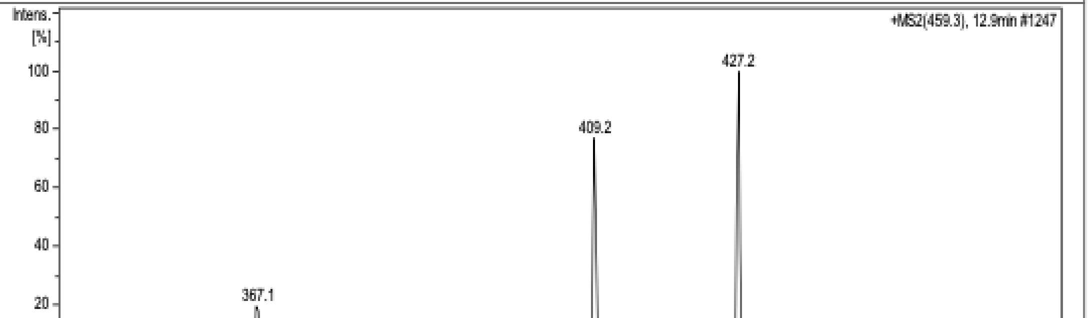

Spectrum Number	11	Detected Mass	227.0599
Adduct Formula	C 7 H 12 N 2 Na O 5		
Putative name	**NAME:** Ala Asp		
Acc #	**Metlin:** 23907<br>**HMDB:**x<br>**KEGG:**x		
Interpretation			
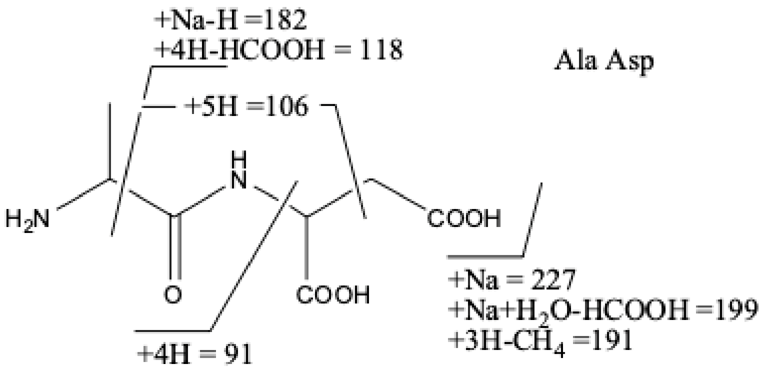			
Product ion spectrum			
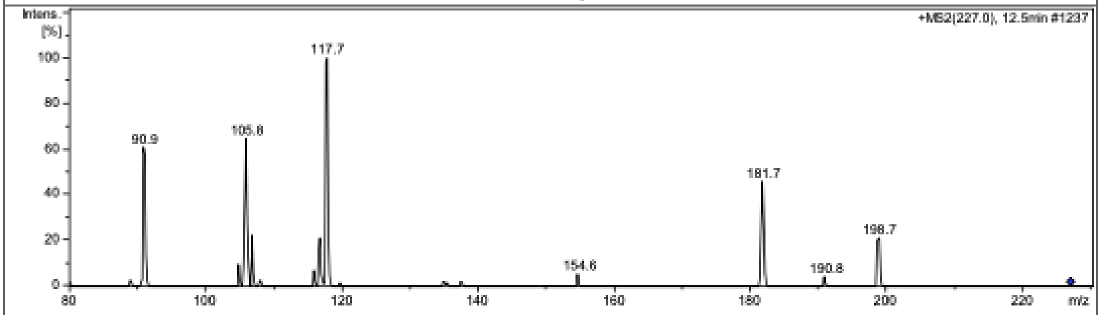			
