# Supplementary material for: Urinary Metabolomic Analysis of Prostate Cancer by UPLC-FTMS and UPLC-Ion Trap MSn
Source: Diagnostics (Basel). 2023 Jul 4;13(13):2270. doi: 10.3390/diagnostics13132270 (PMC10340632; doi:10.3390/diagnostics13132270)

Supplementary Table S3. The product ion spectra of identified metabolites and their authentic standards

1	name	D-mannitol,(m/z 205.0685,)
RT	2.2 min	
Interpretation		
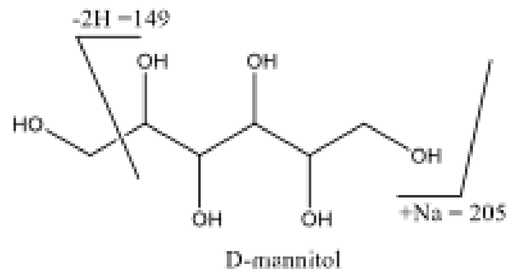		
Product ion spectrum of m/z 205.0 ion		
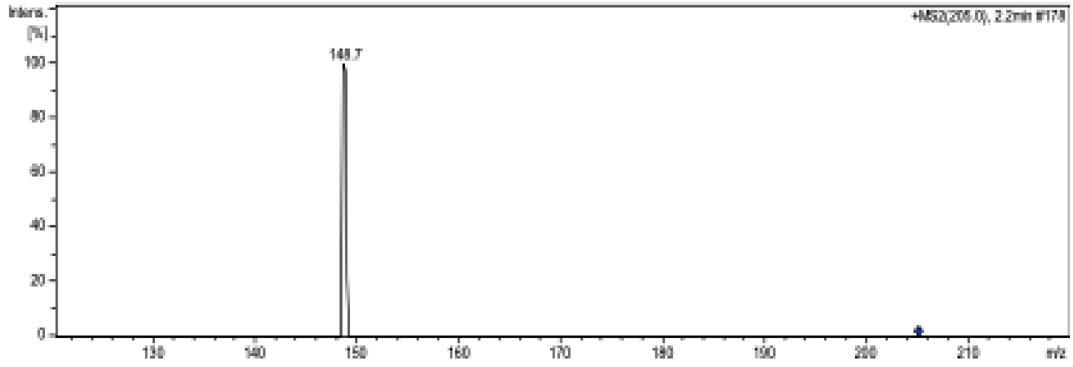		
Product ion spectrum of standard		
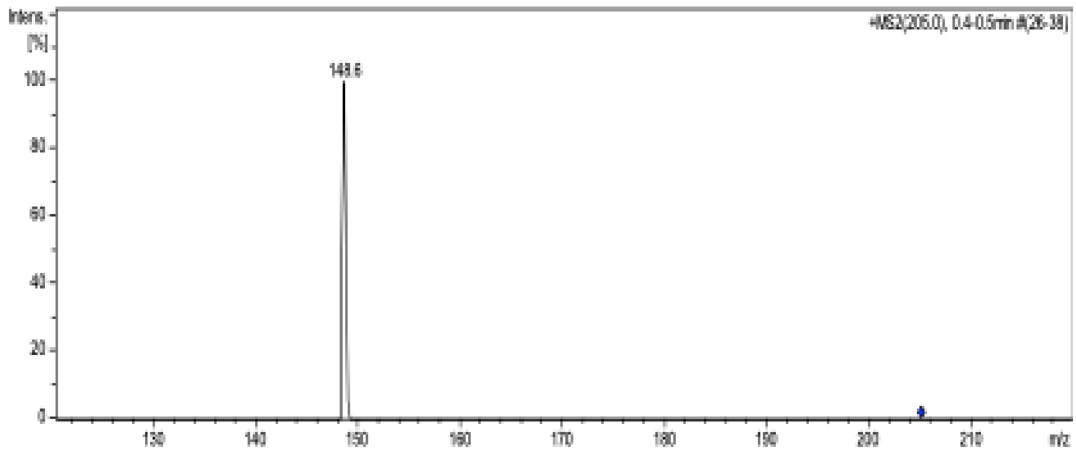		

2	name	N2, N2-dimethylguanosine, (m/z 334.1126)

RT	10.3 min

Interpretation

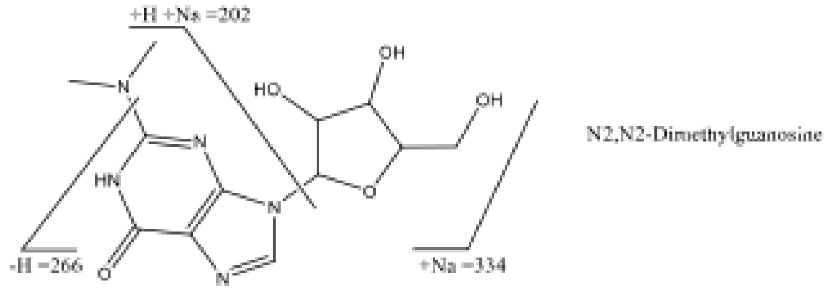

Product ion spectrum of m/z 334.1 ion

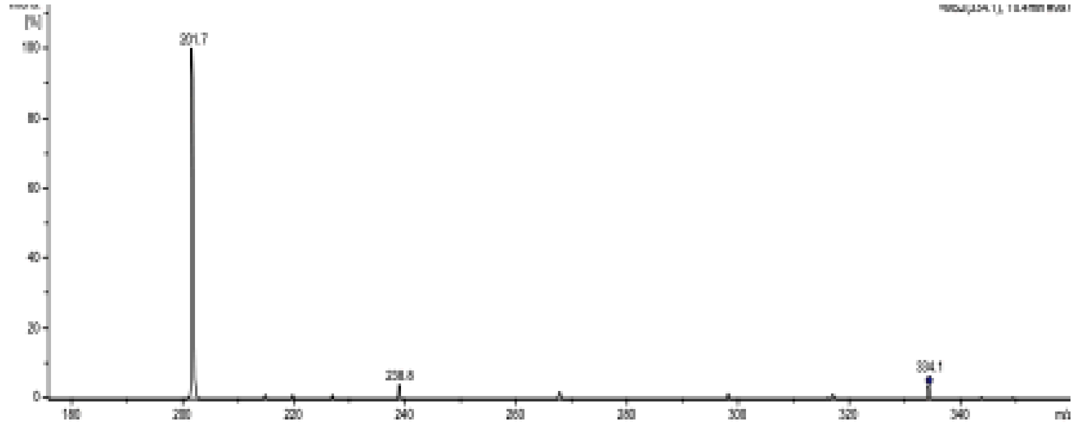

Product ion spectrum of standard

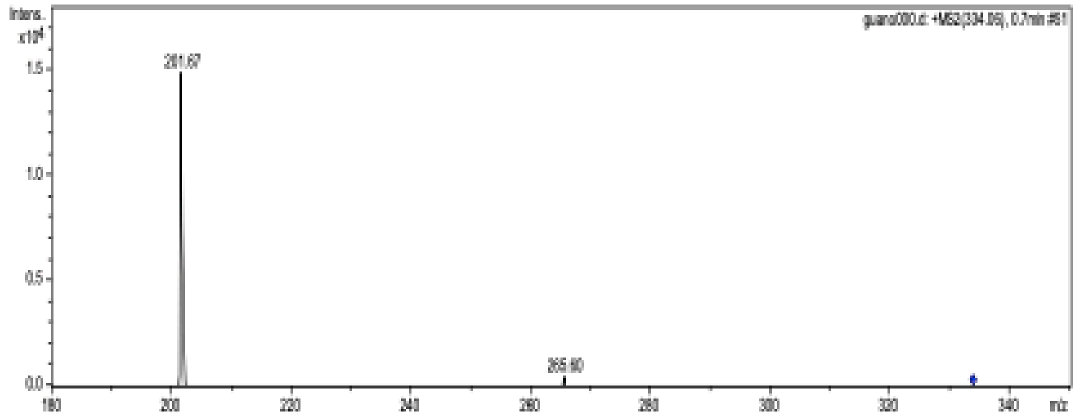

3	name	phenylacetyl L-glutamine(m/z 551.2124)
RT	12.1 min	

Interpretation

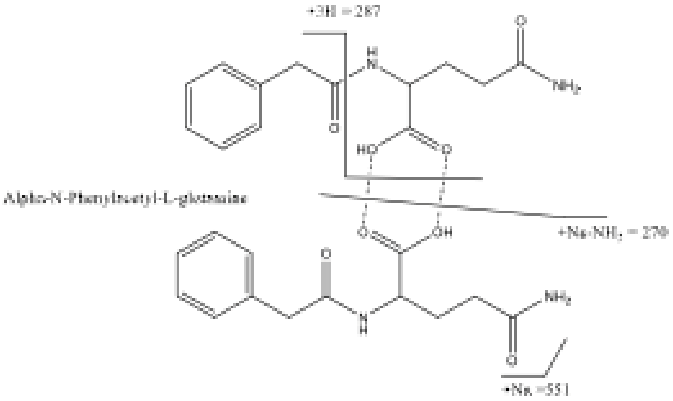

Product ion spectrum of m/z 551.1 ion

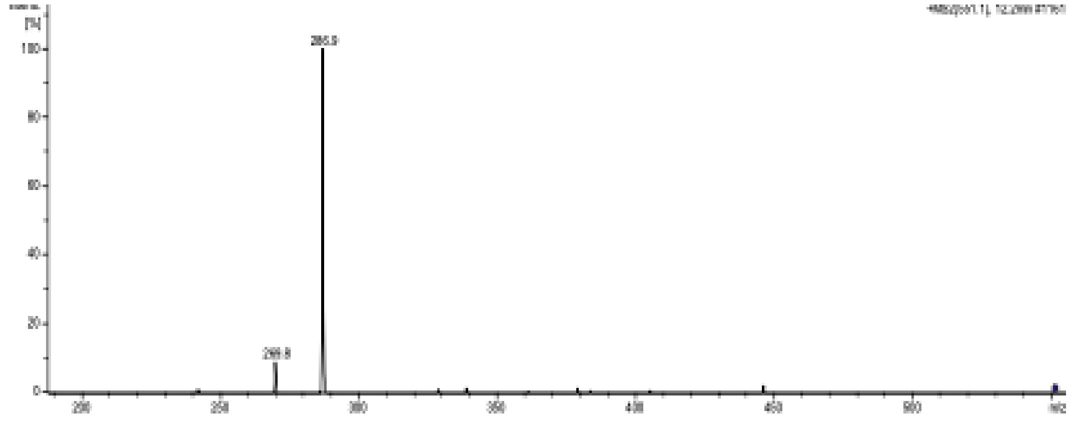

Product ion spectrum of standard

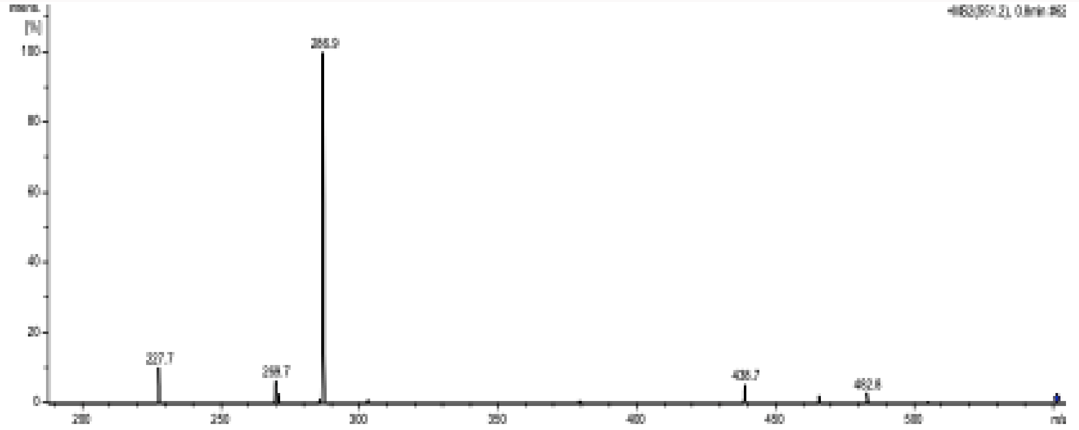

<table>
<tr><td>4</td><td>name</td><td>7-ketodeoxycholic acid(m/z 407.2798)</td></tr>
<tr><td>RT</td><td colspan="2">16.8 min</td></tr>
<tr><td colspan="3">Interpretation</td></tr>
<tr><td colspan="3">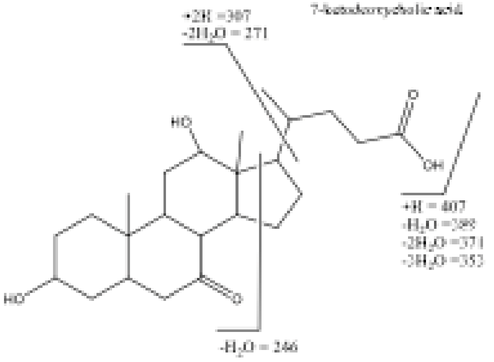
</td></tr>
<tr><td colspan="3">Product ion spectrum of m/z 407.2 ion</td></tr>
<tr><td colspan="3">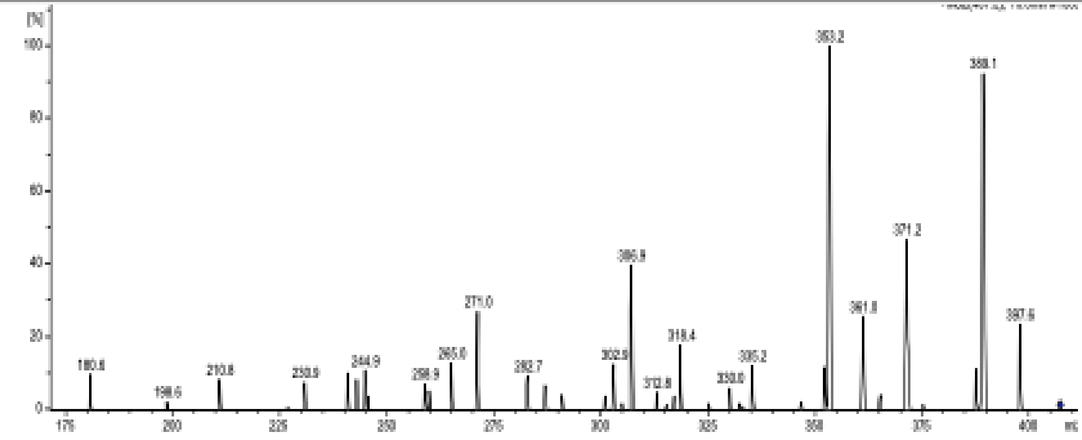
</td></tr>
<tr><td colspan="3">Product ion spectrum of standard</td></tr>
<tr><td colspan="3">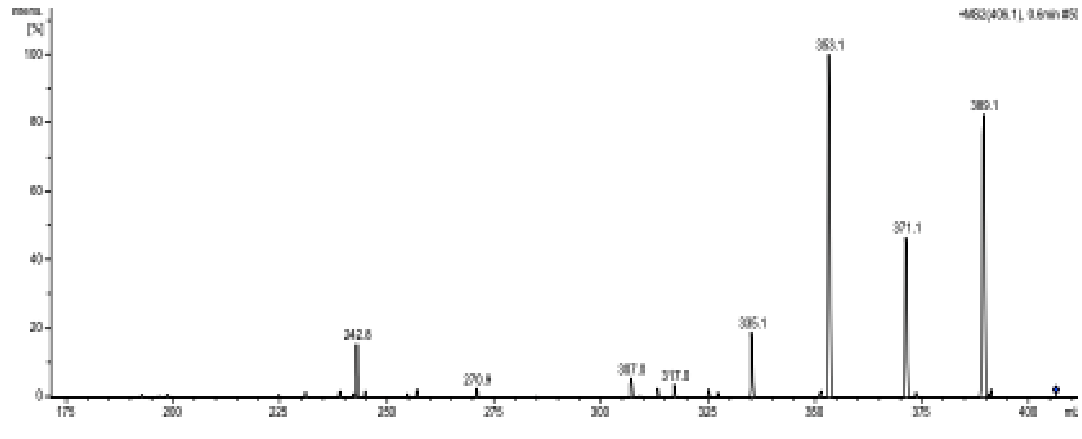
</td></tr>
</table>

5	name	Ala Asp(m/z 227.0599)
RT	12.5 min	

Interpretation

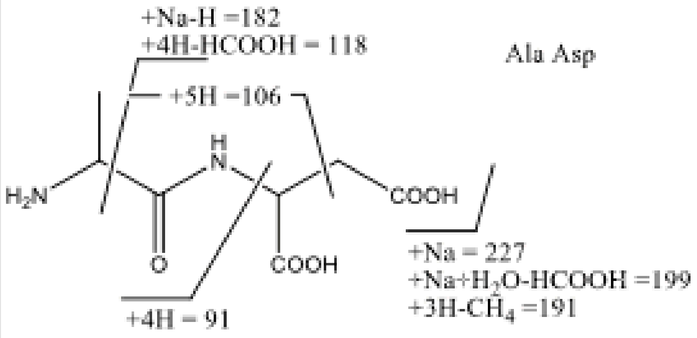

Product ion spectrum of m/z 227.1 ion

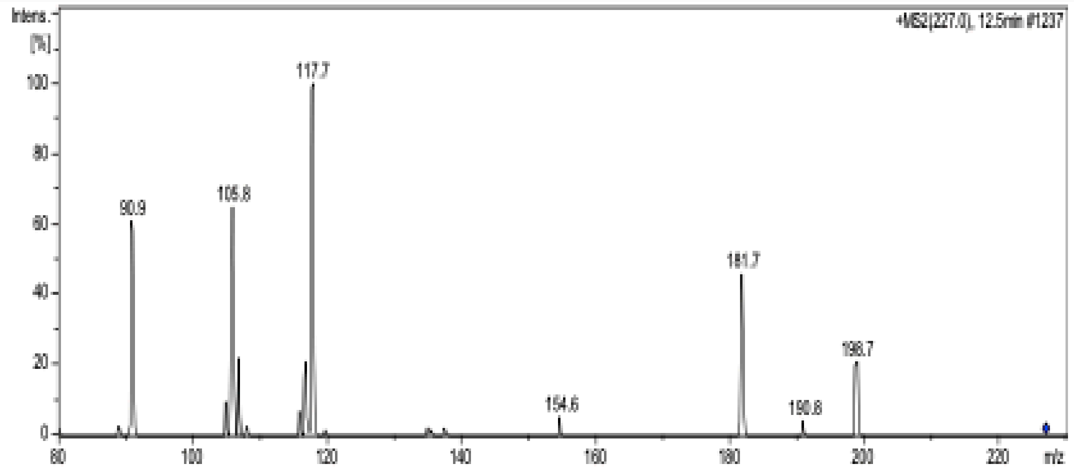

Product ion spectrum of standard

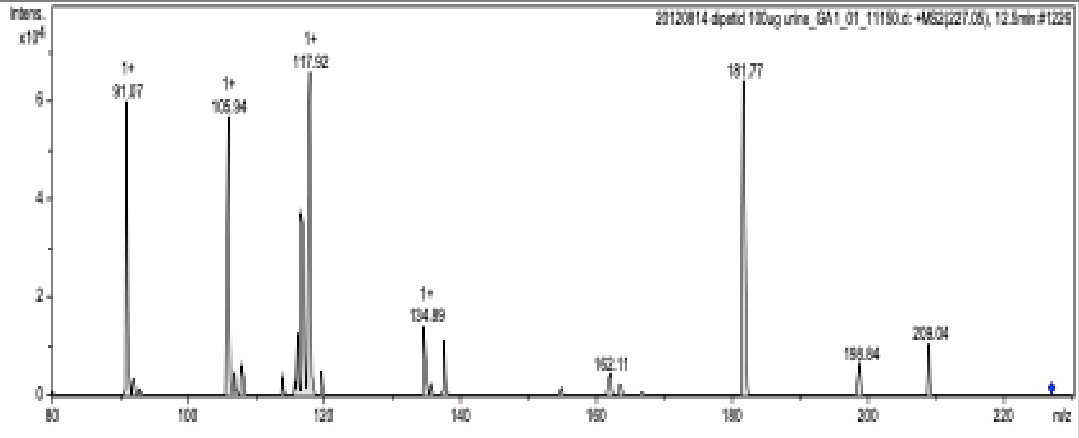

Supplement: Supplementary file 1 [file diagnostics-13-02270-s001.zip › diagnostics-2454829-Table S3.pdf]
